# Efficacy and safety of Chinese patent medicine for urinary tract infections: a systematic review and network meta-analysis

**DOI:** 10.3389/fmed.2025.1622999

**Published:** 2025-08-21

**Authors:** Jialei Zhao, Haibin Tang, Rutong Xu, Gang Chen

**Affiliations:** Department of Urology, The First Affiliated Hospital of Chongqing Medical University, Chongqing, China

**Keywords:** network meta-analysis, randomized controlled trials, urinary tract infections, Chinese medicine, systematic review

## Abstract

**Introduction:**

Chinese patent medicines (CPMs) have garnered increasing attention as therapeutic options for urinary tract infections (UTIs); however, robust evidence supporting their efficacy remains limited. To address this gap, we performed a network meta-analysis (NMA) incorporating both direct and indirect comparisons to systematically evaluate and rank the efficacy and safety profiles of CPMs for UTIs.

**Methods:**

We systematically searched five electronic databases from their inception through April 2025. Data were analyzed using a frequentist approach with random-effects models.

**Results:**

Twenty-three studies with nine types of medicines and 3,250 patients were included in this study. While network meta-analysis suggested that Xueniaoan capsule with antibiotics (XNA_Ab) had the highest probability of being optimal, this conclusion requires cautious interpretation because of the low certainty of evidence from the included studies.

**Conclusion:**

Several CPMs demonstrate potential therapeutic benefits for UTIs; however, these preliminary findings require validation through rigorously designed large-scale randomized controlled trials.

## 1 Introduction

Urinary tract infections (UTIs) are among the most common bacterial infections (1). Worldwide, 150 million patients are diagnosed with UTIs each year. The overall pooled incidence of urinary tract infections was 1.6%, with the highest incidence rate observed in the African region at 3.7% (2). UTIs can lead to septic shock, ranking third among all diseases that can cause death due to infection (3). UTIs can be classified based on the site of infection (lower or upper urinary tract) and the complexity of the host condition (uncomplicated or complicated), resulting in categories such as lower or upper UTI and complicated or uncomplicated UTI. These infections can present with or without symptoms (4). The incidence and global burden of urinary tract infections have remained relatively stable over the past three decades, yet the associated mortality rate has shown an annual increase of 0.55%. By 2019, global UTI-related deaths had reached 2.4 times the number recorded in 1990. The disease burden exhibits an age-dependent progression among elderly populations, with a particularly marked upward trend observed in individuals aged > 60 years. Patients with diabetes mellitus demonstrate significantly higher susceptibility to urinary tract infections (UTIs), with treatment costs exceeding those for non-diabetic patients by a factor of 1.20–1.50 (5). Given the ongoing demographic aging and population growth, the global burden of UTIs is projected to intensify without appropriate intervention measures (6).

Urine specimens are most frequently submitted for culturing in clinical microbiology laboratories. UTIs can be caused by infections from various microorganisms, with significant variations observed in the epidemiology, species distribution, and susceptibility patterns of urinary pathogens across different populations and geographic locations (7). At present, antibiotics are the main treatment for UTIs; however, the misuse of antibiotics significantly increases bacterial resistance, reduces the clinical efficacy of antibiotics, increases the recurrence rate of bacterial infections, and wastes medical resources. These treatments carry the potential to permanently alter the normal microbiota of both the gastrointestinal and vaginal tracts while promoting the proliferation of multidrug-resistant microorganisms. Crucially, the “golden era” of antibiotics is approaching its end (8). The global inappropriate use and overuse of antibiotics have led to the worldwide spread of multidrug-resistant (MDR) and pandrug-resistant (PDR) bacterial strains, commonly known as “superbugs” (9, 10). Some studies indicate that low- and middle-income countries are experiencing significantly higher rates of antimicrobial resistance escalation compared to high-income countries (11). For Gram-negative bacteria, substantial research efforts have focused on understanding and improving treatment strategies against drug-resistant strains (12). A World Health Organization (WHO) study demonstrated that antibiotic resistance is directly responsible for over 730,000 deaths worldwide annually. Herbal and natural medicines may represent an effective therapeutic alternative for multidrug-resistant urinary tract infection (UTI) pathogens. Traditional plant-based therapies have been widely used for disease management throughout history, with approximately 80% of the population in developing nations currently dependent on these natural remedies. Natural antimicrobial agents can be derived from diverse sources, including animal tissues, microorganisms, and various plant materials such as bark, stems, leaves, flowers, and fruits (13). Natural medicines demonstrate fewer adverse side effects while offering superior therapeutic benefits. Research has confirmed that most Gram-positive and Gram-negative uropathogens—including *Escherichia coli*, *Klebsiella*, and *Proteus species*—can be effectively controlled and alleviated through natural medicinal interventions (14).

Traditional Chinese Medicine (TCM) has become a fully institutionalized component of China’s healthcare system. Current estimates indicate that TCM providers deliver medical services to over 200 million outpatients and 7 million inpatients annually, accounting for approximately 10–20% of all patients in China (15). Chinese Patent Medicines (CPM) are standardized herbal formulations produced with specific dosages and administration methods, representing a commercialized form of Traditional Chinese Medicine that has been approved by China’s National Medical Products Administration. With advancements in pharmaceutical technology, over 10,000 distinct CPM formulations have been developed to date (16). CPMs typically consist of multiple herbal ingredients, with each component containing distinct chemical constituents that act on different therapeutic targets through diverse pharmacological pathways to exert their therapeutic effects (17). CPMs play a vital role in the treatment, prevention, and rehabilitation of various diseases, with extensive clinical data and evidence supporting their efficacy (18). Having gained widespread acceptance in China, these medicines are increasingly being adopted as complementary therapies in the European and North American markets (19, 20). The effects of clearing heat and toxicity, removing dampness, treating stranguria, and tonifying the kidney are often used for the treatment of hot stranguria; reddish urine; and the painful, urgent, and frequent urination caused by damp heat in the lower jiao, as well as acute or chronic pyelonephritis, cystitis, and UTIs with these symptoms and signs (17). A variety of CPMs have been shown to have bacteriostatic effects on pathogenic microorganisms; therefore, they may inhibit or destroy the formation of toxic substances. This study used network meta-analysis (NMA) to systematically evaluate the efficacy and safety of CPMs in the treatment of UTIs to provide more reference for the treatment of UTIs and the application of CPMs.

## 2 Methods

This meta-analysis of RCTs was performed in accordance with the PRISMA statement and registered with the PROSPERO International Prospective Register of Systematic Reviews (registration number CRD42023455364).

### 2.1 Inclusion criteria

Studies were selected according to the following inclusion criteria:

1. Participants were diagnosed with UTIs
  - A mid-stream sample of urine showing bacterial growth > 10^5^ cfu/mL;
  - ≥10 white blood cells/mm^3^ of unspun urine;
  - At least one of the following symptoms: frequency or urgency, dysuria, suprapubic tenderness, or costovertebral angle pain or tenderness (not explained by other diagnoses).
2. This study was performed as a randomized controlled trial.
3. These drugs used are Chinese patent medicines produced in a standardized manner.
4. The outcomes included the following indicators:
  - Cure rate: the symptoms disappeared, and the leukocyte levels in the urine returned to normal after treatment
  - Total effective rate: the symptoms partially disappeared or the value of urine leukocytes was reduced, but did not return to normal after treatment
  - Bacterial clearance rate: the original infected part of the specimen did not regenerate after treatment
  - Adverse events included headache, stomach ache, stomach discomfort, mild nausea, skin rash, and dizziness
5. No language restrictions were imposed.

### 2.2 Exclusion criteria

The exclusion criteria were as follows: (1) duplicated data; (2) the full text was not available; (3) insufficient data (miscalculation or missing data); and (4) the intervention included other Chinese drugs, acupuncture, and massage (including a proprietary Chinese drug, Traditional Chinese Medicine extract injection, decoction, auricular points, and other Traditional Chinese Medicine methods as auxiliary treatment).

### 2.3 Search strategy

We searched articles with no language restriction in five electronic databases, including PubMed, EMBASE, Ovid Medline, China National Knowledge Infrastructure (CNKI), and WanFang Database, and systematically searched for entries added between inception and April 2025. The following search terms were used separately or in combination: “Traditional Chinese Medicine” and “urinary tract infections.” The search strategies for databases are presented in Supplementary Appendix A.

### 2.4 Literature screening and data extraction

Two authors independently extracted the data. Any disagreement will be resolved by discussion until a consensus is reached or by consulting a third author. The following data were extracted: author, year of publication, study period, original inclusion criteria, total number of patients included in the study, doses of medicines, time of application, cure rate, total effective rate, bacterial clearance rate, and incidence of adverse events.

### 2.5 Risk of bias assessment and quality assessment

The risk of study bias was assessed by two independent reviewers using the Cochrane risk of bias (RoB 2) tool. Each included study used the RoB 2 tool to evaluate literature quality, including the randomization process, deviations from intended interventions, missing outcome data, measurement of the outcome, selection of the reported result, and overall bias. Each RCT was classified as having high, low, or unclear risk for the above items. If one of the items was classified as high risk, the overall quality was classified as high risk.

Two researchers independently assessed the quality of evidence using the GRADE framework, considering the risk of bias, inconsistency, indirectness, imprecision, and publication bias (21).

### 2.6 Statistical analysis

Statistical analyses were performed using R software—version 4.3.1. All statistical methods in this network are based on a Bayesian framework. Since there were certainly potential differences between studies, we chose a random-effects model for our analysis rather than a fixed-effects model. The model uses four Markov chains for the initial value setting. The initial value is set to 2.5, pre-iterated 10,000 times for annealing, and iterated 50,000 to achieve model convergence, drawing a trajectory density map and convergence diagnostic map to determine whether the model fits. When the Potential Scale Reduction Factor (PSRF) tends to 1, the model convergence is satisfactory; otherwise, the number of iterations continues to increase. Relative risk (RR) and 95% confidence interval (CI) were used for binary outcomes, and weighted mean difference and 95% CIs were used for continuous outcomes. The consistency was tested using the node-splitting method. Meanwhile, the hierarchy of treatment rankings was estimated by the value of the surface under the cumulative ranking curve (SUCRA), and the larger SUCRA value of comparisons was regarded as superior efficacy or lower incidence of adverse events.

Stata 16.0 software was used to present and describe the network diagram of the different exercise interventions. In the generated network diagram, each node represents a different motor intervention and control condition, and the lines connecting the nodes represent a direct positive comparison between the interventions. The size of each node and width of the connecting lines were proportional to the number of studies.

## 3 Results

### 3.1 Study selection and characteristics

**Figure 1:**
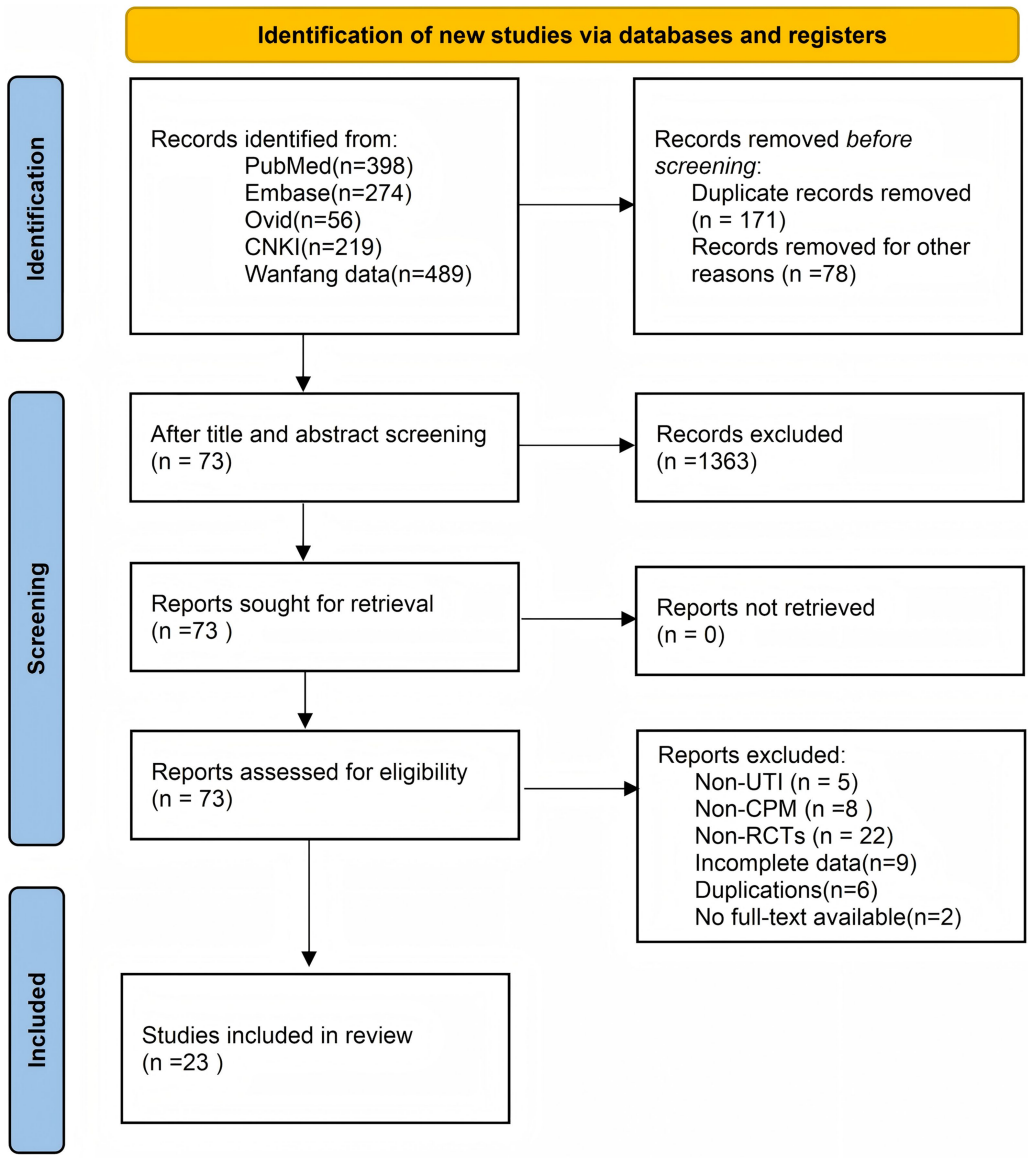
Flowchart of literature screening.

A preliminary literature search identified 1,508 included articles [PubMed (*n* = 398), Embase (*n* = 274), Ovid Medline (*n* = 56), CNKI (*n* = 219), and Wanfang data (*n* = 489)]. During our initial search, we excluded irrelevant articles that did not meet the inclusion requirements, such as animal studies, non-UTI studies, non-proprietary Chinese medicine studies, duplicate articles, and reviews. After filtering by title, 151 articles were downloaded for further screening. Publications that did not meet the inclusion criteria and were duplicates were excluded. The selection process for this study is shown in Figure 1.

A total of 23 papers were included in this study, covering nine Chinese patent medicines and 3,250 patients (22–44). The intervention measures in the trial group included nine medicines: Yinhua Miyanling tablet, Xueniaoan capsule, Sanjin tablet, Shuangdong capsule, Longqing tablet, Ningmitai capsule, Bixiefenqing pill, Niaotongkakenaiqi tablet, and Compound Shiwei tablet. The control group was treated with antibiotics (including levofloxacin hydrochloride, aztreonam, Cefdinir, Ceftazidime, and moxifloxacin). The treatment course ranged from 7 to 42 weeks (Table 1).

**Table 1:** Characteristics of included RCTs.

First author (year)	Group	Sample size	Age	Male	Female	Interventions	Durations/d	Outcomes
Yuan 2024	G1	29	39.65 ± 2.68(22 ~ 64)	10	19	YHMYL+Ab	10	a.b
	G2	29	39.78 ± 2.74(23 ~ 63)	11	18	Ab	10	a.b
Pan 2022	G1	60	47.16 ± 5.06(42 ~ 60)	0	60	XNA + Ab	14	a.b.c.d
	G2	60	48.16 ± 4.02(40 ~ 59)	0	60	Ab	14	a.b.c.d
Cui 2022	G1	77	32.2 ± 9.40	24	53	SJP	7	a.c.d
	G2	79	32.72 ± 9.97	23	56	Ab	7	a.c.d
	G3	77	32.38 ± 9.7	23	54	SJP + Ab	7	a.c.d
Wang 2022	G1	47	73.29 ± 2.38(63 ~ 81)	20	27	SD + Ab	14	a.b.d
	G2	46	72.57 ± 3.02(62 ~ 80)	21	25	Ab	14	a.b.d
Chen 2022	G1	50	65.34 ± 1.89(62 ~ 79)	22	28	LQ + Ab	14	b
	G2	50	65.68 ± 2.21(61 ~ 78)	23	27	Ab	14	b
Hu 2021	G1	40	44.5 ± 8.25(28 ~ 70)	8	32	XNA + Ab	14	a.b.c.d
	G2	40	43.85 ± 9.97(26 ~ 72)	9	31	Ab	14	a.b.c.d
Duan 2019	G1	124	44.19 ± 11.37(25 ~ 61)	64	60	SJP + Ab	7	a.b.d
	G2	124	44.03 ± 10.43(24 ~ 62)	60	64	Ab	7	a.b.d
Ou 2019	G1	42	45.38 ± 1.39(20 ~ 70)	22	20	XNA + Ab	14	a.b.c.d
	G2	42	45.13 ± 1.25(20 ~ 68)	24	18	Ab	14	a.b.c.d
Wang 2018	G1	54	66 ± 6	0	54	YHMYL+Ab	14	a.b.d
	G2	54	66 ± 7	0	54	Ab	14	a.b.d
Chen 2018	G1	53	51.2 ± 3.1(50 ~ 57)	0	53	SJP + Ab	7	a.b
	G2	53	51.1 ± 3.2(48 ~ 56)	0	53	Ab	14	a.b
Dou 2017	G1	26	\	16	20	NMT	14	a.b.d
	G2	10	\			SJP	14	a.b.d
Liu 2017	G1	72	35.9 ± 14.14(18 ~ 75)	22	50	SJP + Ab	7	a.b.c.d
	G2	71	39.14 ± 15.82(18 ~ 73)	27	44	Ab	7	a.b.c.d
	G3	70	39.7 ± 14.37(20 ~ 74)	28	42	SJP	7	a.b.c.d
Yang 2015	G1	37	35.7 ± 8.2(18 ~ 65)	6	31	BXFQ+Ab	14	a.b.c.d
	G2	37	41.5 ± 8.2(23 ~ 68)	5	32	Ab	14	a.b.c.d
Lu 2015	G1	63	46.3 ± 10.8(20^65)	30	33	NMT + Ab	15	a.b.c.d
	G2	63	45.2 ± 11.3(23 ~ 68)	27	36	Ab	15	a.b.c.d
Sun 2014	G1	319	\	\	\	NTKKNQ	7	a.b.d
	G2	107	\	\	\	SJP	7	a.b.d
Lyu 2014	G1	61	62.54 ± 9.88(20 ~ 70)	10	51	NMT	14	a.b.c.d
	G2	34	64.03 ± 9.1(20 ~ 70)	5	29	SJP	14	a.b.c.d
Lyu 2012	G1	56	41.64 ± 13.97	1	55	NTKKNQ	7	a.b
	G2	20	43.11 ± 14.22	0	20	SJP	7	a.b
Kong 2011	G1	30	35.5 ± 11(18 ~ 63)	10	20	SJP + Ab	14	a.b
	G2	28	36.44 ± 11.3(19 ~ 65)	9	19	Ab	14	a.b
Sun 2008	G1	113	66.2(51 ~ 83)	50	63	LQ	28	a.b.c
	G2	110	66.2(51 ~ 83)	37	73	Ab	28	a.b.c
Wang 2008	G1	81	42.79 ± 11.94	9	72	FFSW	7	a.b
	G2	27	48.52 ± 11.27	24	3	SJP	7	a.b
Zhan 2007	G1	110	46.26 ± 12.57	30	80	FFSW	42	a.b.d
	G2	37	46.68 ± 14.40	9	28	SJP	42	a.b.d
	G1	206	44.18 ± 12.28	21	185	FFSW	7	a.b.d
	G2	106	46.12 ± 11.45	10	96	SJP	7	a.b.d
Liu 2006	G1	55	\	7	48	FFSW	7	a.b.d
	G2	53	\	7	46	SJP	7	a.b.d
Zhang 2004	G1	44	35 ~ 78	27	61	NMT	14	a.b
	G2	44				Ab	14	a.b

a. cure rate; b. effective rate; c. bacterial clearance; d. adverse events; YHMYL, Yinhua Miyanling tablet; XNA, Xueniaoan capsule; SJT, Sanjin tablet; SD, Shuangdong capsule; LQ, Longqing tablet; NMT, Ningmitai capsule; BF, Bixiefenqing pill; NTKKNQ, Niaotongkakenaiqi tablet; FFSW, Compound Shiwei tablet; Ab, conventional antibiotics. Effect sizes where statistical differences exist are bolded.

### 3.2 Quality assessment and quality assurance

**Figure 2:**
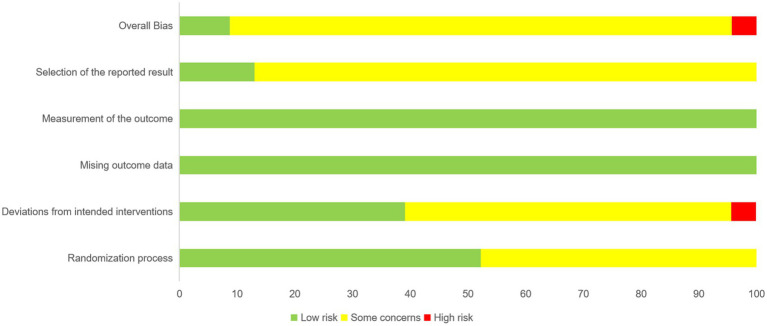
Risk of bias graph indicating the review authors’ rating regarding the risk of bias across all included studies.

The results of the quality assessment are presented in Figure 2. Some studies in the randomization process showed that allocation concealment was not adequately described. In deviations from the intended interventions, blinding of subjects and implementers could not be ensured in the study, and there was no description of the study setting. One study did not describe a baseline comparison of RCT studies and was considered to be at high risk. The main problem with the selection of the reported results was that there was no verifiable evidence that the actual analysis was consistent with the predesign. Supplementary Appendix B details the assessments of GRADE certainty of evidence for direct, indirect, and network comparisons.

### 3.3 Network diagram

**Figure 3:**
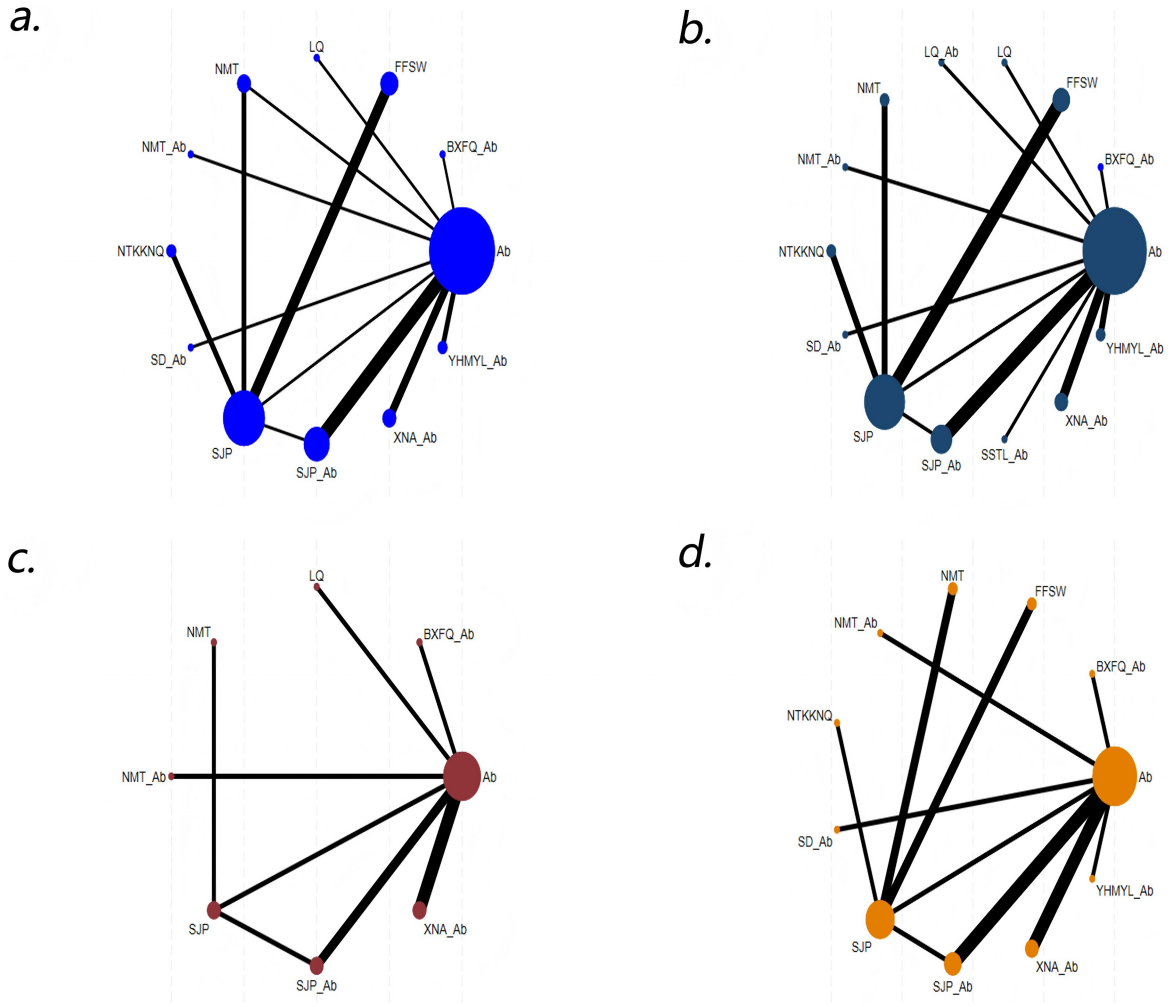
Network diagram: **(a)** cure rate; **(b)** effective rate; **(c)** bacterial clearance; **(d)** adverse events.

A total of 23 RCTs with nine participants were included in the study. A line between the two points represents evidence of a direct comparison between two CPMs, and the thickness of the line indicates the number of RCTs. The absence of a line between the two points indicates that no RCTs reported direct comparisons between the two drugs or indirectly compared the efficacy of the two drugs (Figure 3).

### 3.4 Network meta-analysis

#### 3.4.1 Cure rate

**Figure 4:**
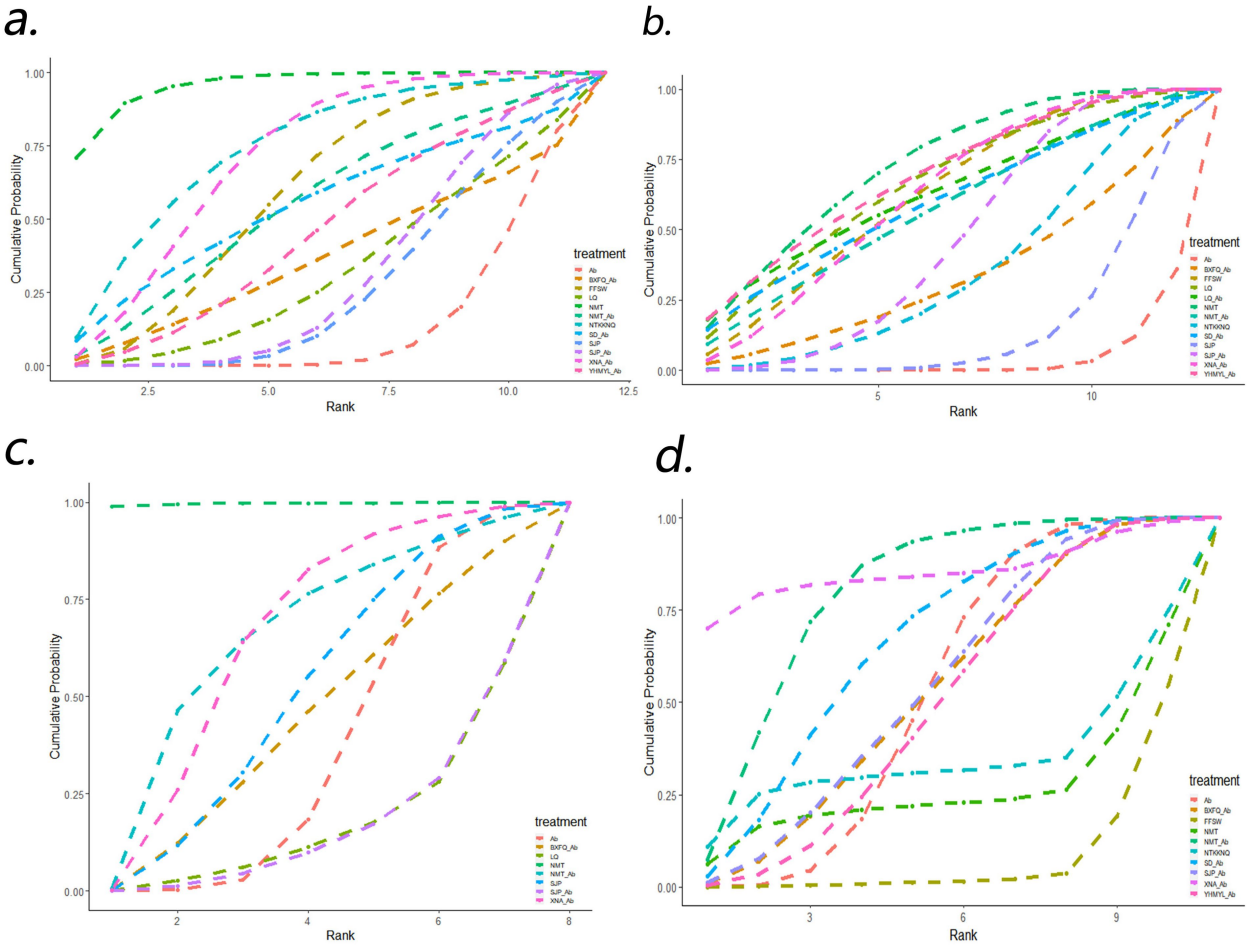
Rank plots: **(a)** cure rate; **(b)** effective rate; **(c)** bacterial clearance; **(d)** adverse events. BXFQ_Ab, Bixiefenqing pill combined with antibiotics; FFSW, Compound Shiwei tablet; LQ, Longqing tablet; LQ_Ab, Longqing tablet combined with antibiotics; NMT, Ningmitai capsule; NMT_Ab, Ningmitai capsule combined with antibiotics; NTKKNQ, Niaotongkakenaiqi tablet; SD_Ab, Shuangdong capsule combined with antibiotics; SJT, Sanjin tablet; SJT_Ab, Sanjin tablet combined with antibiotics; XNA_Ab, Xueniaoan capsule combined with antibiotics; YHMYL_Ab, Yinhua Miyanling tablet combined with antibiotics; Ab, conventional antibiotics.

Cure rates were reported in 22 RCTs, including 9 CPM and antibiotics. Compared with antibiotics alone, Ningmitai capsule (RR = 3.44, 95%Cl:1.77 ~ 7.31) and Xueniao’an an capsule combined with antibiotics (RR = 1.86, 95% Cl: 1.23 ~ 3.03) had higher cure rates for UTIs, and there were significant statistical differences between them. Compared with different CPMs, the cure rate associated with Ningmitai capsule was higher than that with Compound Shiwei tablet (RR = 2.15, 95% CI:1.01 ~ 5.03), Longqing tablet (RR = 2.96, 95% CI:1.18 ~ 7.99), Sanjin tablet (RR = 3.03, 95% CI:1.57 ~ 6.51), Sanjin tablet combined with antibiotics (RR = 2.94, 95% CI:1.45, 6.42), and Yinhua Miyanling tablet (RR = 2.52, 95% CI:1.01 ~ 6.51). The overall heterogeneity *I*^2^ = 43.84. The top three SUCRA rankings were Ningmitai capsule (95.72%), Niaotongkakenaiqi tablet (74.22%), and Xueniao’an capsule combined with antibiotics (71.41%). The specific results are shown in Figure 4a and Table 2.

**Table 2:** League table (cure rate).

	Ab	BXFQ_Ab	FFSW	LQ	NMT	NMT_Ab	NTKKNQ	SD_Ab	SJP	SJP_Ab	XNA_Ab	YHMYL_Ab
Ab	Ab	1.21 (0.47, 3.19)	1.6 (0.9, 2.82)	1.17 (0.6, 2.25)	**3.44 (1.77, 7.31)**	1.56 (0.73, 3.4)	2.05 (0.93, 4.39)	1.57 (0.55, 4.84)	1.14 (0.74, 1.72)	1.17 (0.86, 1.63)	**1.86 (1.23, 3.03)**	1.38 (0.76, 2.54)
BXFQ_Ab	0.83 (0.31, 2.13)	BXFQ_Ab	1.32 (0.43, 3.93)	0.97 (0.31, 3.02)	2.85 (0.88, 9.46)	1.3 (0.38, 4.35)	1.7 (0.48, 5.62)	1.31 (0.31, 5.53)	0.94 (0.32, 2.6)	0.97 (0.35, 2.62)	1.55 (0.54, 4.49)	1.14 (0.37, 3.5)
FFSW	0.63 (0.35, 1.12)	0.76 (0.25, 2.35)	FFSW	0.73 (0.3, 1.74)	**2.15 (1.01, 5.03)**	0.98 (0.38, 2.56)	1.28 (0.59, 2.68)	0.99 (0.3, 3.48)	0.71 (0.48, 1.04)	0.73 (0.41, 1.33)	1.16 (0.58, 2.51)	0.86 (0.38, 2.02)
LQ	0.86 (0.44, 1.66)	1.03 (0.33, 3.27)	1.37 (0.57, 3.28)	LQ	**2.96 (1.18, 7.99)**	1.34 (0.49, 3.71)	1.76 (0.62, 4.79)	1.35 (0.4, 4.87)	0.98 (0.45, 2.11)	1 (0.48, 2.08)	1.59 (0.74, 3.72)	1.17 (0.49, 2.9)
NMT	**0.29 (0.14, 0.56)**	0.35 (0.11, 1.13)	**0.47 (0.2, 0.99)**	**0.34 (0.13, 0.85)**	NMT	0.45 (0.16, 1.24)	0.59 (0.21, 1.48)	0.46 (0.12, 1.68)	**0.33 (0.15, 0.64)**	**0.34 (0.16, 0.69)**	0.54 (0.23, 1.22)	**0.4 (0.15, 0.99)**
NMT_Ab	0.64 (0.29, 1.36)	0.77 (0.23, 2.66)	1.02 (0.39, 2.63)	0.75 (0.27, 2.02)	2.21 (0.8, 6.32)	NMT_Ab	1.31 (0.43, 3.78)	1.01 (0.28, 3.87)	0.73 (0.3, 1.72)	0.75 (0.33, 1.72)	1.19 (0.51, 2.98)	0.88 (0.33, 2.34)
NTKKNQ	0.49 (0.23, 1.08)	0.59 (0.18, 2.09)	0.78 (0.37, 1.68)	0.57 (0.21, 1.63)	1.68 (0.67, 4.72)	0.76 (0.26, 2.34)	NTKKNQ	0.77 (0.21, 3.04)	0.56 (0.29, 1.06)	0.57 (0.27, 1.29)	0.91 (0.39, 2.37)	0.67 (0.26, 1.86)
SD_Ab	0.64 (0.21, 1.83)	0.76 (0.18, 3.19)	1.01 (0.29, 3.39)	0.74 (0.21, 2.53)	2.17 (0.6, 8.05)	0.99 (0.26, 3.55)	1.29 (0.33, 4.81)	SD_Ab	0.72 (0.22, 2.26)	0.74 (0.23, 2.26)	1.19 (0.36, 3.8)	0.87 (0.25, 2.94)
SJP	0.88 (0.58, 1.36)	1.06 (0.38, 3.1)	1.41 (0.96, 2.08)	1.03 (0.47, 2.24)	**3.03 (1.57, 6.51)**	1.38 (0.58, 3.34)	1.8 (0.94, 3.42)	1.39 (0.44, 4.6)	SJP	1.03 (0.68, 1.62)	1.64 (0.93, 3.19)	1.21 (0.59, 2.59)
SJP_Ab	0.86 (0.61, 1.17)	1.03 (0.38, 2.85)	1.37 (0.75, 2.42)	1 (0.48, 2.07)	**2.94 (1.45, 6.42)**	1.34 (0.58, 3.04)	1.75 (0.78, 3.75)	1.35 (0.44, 4.31)	0.97 (0.62, 1.48)	SJP_Ab	1.59 (0.94, 2.85)	1.18 (0.59, 2.36)
XNA_Ab	**0.54 (0.33, 0.81)**	0.64 (0.22, 1.85)	0.86 (0.4, 1.73)	0.63 (0.27, 1.34)	1.84 (0.82, 4.26)	0.84 (0.34, 1.96)	1.1 (0.42, 2.59)	0.84 (0.26, 2.81)	0.61 (0.31, 1.08)	0.63 (0.35, 1.06)	XNA_Ab	0.74 (0.34, 1.53)
YHMYL_Ab	0.73 (0.39, 1.32)	0.88 (0.29, 2.73)	1.17 (0.5, 2.65)	0.85 (0.34, 2.05)	**2.52 (1.01, 6.51)**	1.13 (0.43, 3.06)	1.5 (0.54, 3.9)	1.14 (0.34, 4.06)	0.82 (0.39, 1.69)	0.85 (0.42, 1.7)	1.35 (0.65, 2.97)	YHMYL_Ab

Effect sizes where statistical differences exist are bolded.

#### 3.4.2 Effective rate

Effective rates were reported in 23 RCTs, including 9 CPM and antibiotics. Compared with using antibiotics alone, Compound Shiwei table t(RR = 1.25, 95%Cl: 1.03 ~ 1.48), Longqing tablet (RR = 1.27, 95%Cl: 1.05 ~ 1.55), Ningmitai capsule (RR = 1.29, 95%Cl:1.09 ~ 1.56), Ningmitai capsule combined with antibiotics (RR = 1.23, 95%Cl: 1.00 ~ 1.55), Sanjin tablet combined with antibiotics (RR = 1.19, 95%Cl: 1.08 ~ 1.31), Xueniao’an capsule combined with antibiotics (RR = 1.24, 95%Cl: 1.11 ~ 1.42), and Yinhua Miyanling tablet combined with antibiotics (RR = 1.28, 95%Cl: 1.08 ~ 1.67), had higher effective rates for UTIs, and there are significant statistical differences between them. Compared with different CPMs, the effective rate associated with the Compound Shiwei tablet was higher than that with the Sanjin tablet (RR = 1.16, 95% CI:1.05 ~ 1.30). The effective rate associated with the Ningmitai capsule was higher than that with the Sanjin tablet (RR = 1.21, 95% CI:1.04 ~ 1.46). The overall heterogeneity *I*^2^ = 12.25. The top three SUCRA rankings were Ningmitai capsule (72.87%), Yinhua Miyanling tablet combined with antibiotics (68.89%), and Longqing tablet (66.23%). The specific results are shown in Figure 4b and Table 3.

**Table 3:** League table (effective rate).

	Ab	BXFQ_Ab	FFSW	LQ	LQ_Ab	NMT	NMT_Ab	NTKKNQ	SD_Ab	SJP	SJP_Ab	XNA_Ab	YHMYL_Ab
Ab	Ab	1.13 (0.91, 1.43)	**1.25 (1.03, 1.48)**	**1.27 (1.05, 1.55)**	1.26 (0.98, 1.67)	**1.29 (1.09, 1.56)**	**1.23 (1, 1.55)**	1.14 (0.93, 1.37)	1.24 (0.98, 1.63)	1.07 (0.91, 1.22)	**1.19 (1.08, 1.31)**	**1.24 (1.11, 1.42)**	**1.28 (1.08, 1.67)**
BXFQ_Ab	0.89 (0.7, 1.1)	BXFQ_Ab	1.1 (0.81, 1.45)	1.13 (0.83, 1.51)	1.12 (0.79, 1.6)	1.15 (0.85, 1.53)	1.09 (0.79, 1.49)	1.01 (0.74, 1.34)	1.11 (0.78, 1.55)	0.95 (0.71, 1.22)	1.05 (0.81, 1.34)	1.11 (0.84, 1.42)	1.14 (0.85, 1.6)
FFSW	**0.8 (0.67, 0.97)**	0.91 (0.69, 1.23)	FFSW	1.02 (0.79, 1.35)	1.01 (0.75, 1.43)	1.04 (0.86, 1.29)	0.99 (0.75, 1.34)	0.92 (0.77, 1.08)	1 (0.75, 1.39)	**0.86 (0.77, 0.96)**	0.95 (0.81, 1.16)	1 (0.81, 1.27)	1.03 (0.81, 1.44)
LQ	**0.79 (0.65, 0.95)**	0.89 (0.66, 1.21)	0.98 (0.74, 1.26)	LQ	1 (0.72, 1.39)	1.02 (0.78, 1.33)	0.97 (0.73, 1.3)	0.9 (0.67, 1.16)	0.98 (0.72, 1.36)	0.84 (0.65, 1.06)	0.94 (0.75, 1.16)	0.98 (0.78, 1.23)	1.01 (0.78, 1.39)
LQ_Ab	0.79 (0.6, 1.02)	0.89 (0.63, 1.27)	0.99 (0.7, 1.33)	1 (0.72, 1.39)	LQ_Ab	1.02 (0.73, 1.4)	0.98 (0.68, 1.37)	0.9 (0.64, 1.23)	0.99 (0.68, 1.42)	0.85 (0.61, 1.12)	0.94 (0.7, 1.23)	0.99 (0.73, 1.31)	1.02 (0.73, 1.45)
NMT	**0.77 (0.64, 0.92)**	0.87 (0.65, 1.17)	0.96 (0.78, 1.16)	0.98 (0.75, 1.28)	0.98 (0.71, 1.36)	NMT	0.95 (0.72, 1.26)	0.88 (0.7, 1.07)	0.96 (0.71, 1.32)	**0.83 (0.69, 0.96)**	0.92 (0.76, 1.1)	0.96 (0.77, 1.2)	0.99 (0.77, 1.37)
NMT_Ab	**0.81 (0.65, 1)**	0.91 (0.67, 1.26)	1.01 (0.75, 1.33)	1.03 (0.77, 1.37)	1.02 (0.73, 1.46)	1.05 (0.79, 1.39)	NMT_Ab	0.93 (0.68, 1.22)	1.01 (0.72, 1.43)	0.87 (0.65, 1.11)	0.96 (0.75, 1.21)	1.01 (0.78, 1.3)	1.04 (0.78, 1.46)
NTKKNQ	0.87 (0.73, 1.08)	0.99 (0.75, 1.35)	1.09 (0.92, 1.29)	1.11 (0.86, 1.49)	1.11 (0.81, 1.57)	1.13 (0.93, 1.42)	1.08 (0.82, 1.47)	NTKKNQ	1.09 (0.81, 1.53)	0.94 (0.82, 1.06)	1.04 (0.87, 1.29)	1.09 (0.88, 1.4)	1.13 (0.87, 1.58)
SD_Ab	0.8 (0.61, 1.02)	0.9 (0.64, 1.28)	1 (0.72, 1.34)	1.02 (0.74, 1.39)	1.01 (0.7, 1.47)	1.04 (0.76, 1.41)	0.99 (0.7, 1.39)	0.92 (0.66, 1.24)	SD_Ab	0.86 (0.63, 1.13)	0.95 (0.72, 1.24)	1 (0.74, 1.32)	1.03 (0.75, 1.47)
SJP	0.93 (0.82, 1.1)	1.05 (0.82, 1.41)	**1.16 (1.05, 1.3)**	1.19 (0.95, 1.54)	1.18 (0.89, 1.63)	**1.21 (1.04, 1.46)**	1.15 (0.9, 1.53)	1.07 (0.94, 1.21)	1.17 (0.88, 1.59)	SJP	1.11 (0.98, 1.3)	1.16 (0.97, 1.43)	1.2 (0.96, 1.64)
SJP_Ab	**0.84 (0.76, 0.93)**	0.95 (0.75, 1.23)	1.05 (0.86, 1.24)	1.07 (0.86, 1.33)	1.06 (0.82, 1.43)	1.09 (0.91, 1.32)	1.04 (0.82, 1.33)	0.96 (0.78, 1.15)	1.05 (0.81, 1.4)	0.9 (0.77, 1.02)	SJP_Ab	1.05 (0.9, 1.23)	1.08 (0.88, 1.43)
XNA_Ab	**0.8 (0.7, 0.9)**	0.9 (0.7, 1.19)	1 (0.79, 1.23)	1.02 (0.81, 1.29)	1.01 (0.76, 1.37)	1.04 (0.83, 1.29)	0.99 (0.77, 1.28)	0.92 (0.72, 1.14)	1 (0.76, 1.35)	0.86 (0.7, 1.03)	0.95 (0.81, 1.11)	XNA_Ab	1.03 (0.83, 1.38)
YHMYL_Ab	**0.78 (0.6, 0.93)**	0.88 (0.63, 1.18)	0.97 (0.7, 1.24)	0.99 (0.72, 1.29)	0.98 (0.69, 1.37)	1.01 (0.73, 1.3)	0.96 (0.69, 1.28)	0.89 (0.63, 1.15)	0.97 (0.68, 1.33)	0.83 (0.61, 1.04)	0.93 (0.7, 1.13)	0.97 (0.73, 1.21)	YHMYL_Ab

Effect sizes where statistical differences exist are bolded.

#### 3.4.3 Bacterial clearance

Nine RCTs, containing six CPMs, reported bacterial clearance. Compared with the use of antibiotics alone, Ningmitai capsules (RR = 3.08e+07, 95%Cl:4.83 ~ 1.59e+22) had higher bacterial clearance for UTIs, and there were significant statistical differences between them. The top three SUCRA rankings were Ningmitai capsules (99.61%), Xueniaoan capsules combined with antibiotics (65.73%), and Ningmitai capsules combined with antibiotics (65.53%). The specific results are shown in Figure 4c and Table 4. The initial analysis revealed an extremely wide confidence interval for the NMT. In the sensitivity analysis, after excluding one NMT-related study, no statistically significant differences were observed among the other interventions (Supplementary Appendix C). This extreme value may have resulted from the substantially higher bacterial clearance rate in the NMT treatment group than in the control group (4/5 vs. 0/2) in that particular study, leading to statistical instability.

**Table 4:** League table (bacterial clearance).

	Ab	BXFQ_Ab	LQ	NMT	NMT_Ab	SJP	SJP_Ab	XNA_Ab
Ab	Ab	1.09 (0.32, 3.88)	0.67 (0.2, 2.27)	**3.08e+07 (4.83 ~ 1.59e+22)**	1.63 (0.45, 6.38)	1.17 (0.54, 3.28)	0.68 (0.28, 2.02)	1.47 (0.73, 2.97)
BXFQ_Ab	0.92 (0.26, 3.11)	BXFQ_Ab	0.62 (0.11, 3.4)	**2.82e+07 (4.00 ~ 1.37e+22)**	1.5 (0.25, 9.42)	1.07 (0.26, 5.42)	0.62 (0.14, 3.37)	1.35 (0.31, 5.53)
LQ	1.48 (0.44, 5.1)	1.62 (0.29, 9.25)	LQ	**4.53e+07 (4.57 ~ 2.29e+22)**	2.41 (0.41, 15.02)	1.73 (0.45, 8.83)	1 (0.24, 5.24)	2.18 (0.52, 9)
NMT	**0 (0, 0.21)**	**0 (0, 0.25)**	**0 (0, 0.15)**	NMT	**0 (0, 0.39)**	**0 (0, 0.24)**	**0 (0, 0.15)**	**0 (0, 0.3)**
NMT_Ab	0.61 (0.16, 2.24)	0.67 (0.11, 3.98)	0.41 (0.07, 2.42)	**1.95e+07 (2.58 ~ 9.10e+22)**	NMT_Ab	0.72 (0.16, 3.94)	0.41 (0.09, 2.4)	0.9 (0.2, 3.88)
SJP	0.85 (0.3, 1.84)	0.93 (0.18, 3.9)	0.58 (0.11, 2.23)	**2.62e+07 (4.10 ~ 1.27e+22)**	1.39 (0.25, 6.29)	SJP	0.58 (0.22, 1.49)	1.26 (0.35, 3.51)
SJP_Ab	1.47 (0.5, 3.58)	1.62 (0.3, 7.35)	1 (0.19, 4.1)	**4.53e+07 (4.83 ~ 1.6e+22)**	2.41 (0.42, 11.76)	1.74 (0.67, 4.62)	SJP_Ab	2.16 (0.57, 6.67)
XNA_Ab	0.68 (0.34, 1.38)	0.74 (0.18, 3.2)	0.46 (0.11, 1.92)	**2.09e+07 (3.29 ~ 1.05e+22)**	1.11 (0.26, 5.11)	0.79 (0.28, 2.83)	0.46 (0.15, 1.75)	XNA_Ab

Effect sizes where statistical differences exist are bolded.

#### 3.4.4 Adverse events

A total of 15 RCTs containing 9 CPMs reported adverse events. Compared with antibiotics alone, Compound Shiwei tablets (RR = 149603.76, 95%Cl: 1.86 ~ 7,654,678,491,827,824) had higher adverse events for UTIs, and there were significant statistical differences between them. Compared with different CPMs, the adverse events associated with Compound Shiwei tablet was higher than that with Bixiefenqing pill combined with antibiotics (RR = 163612.61, 95%Cl: 1.53 ~ 7,149,846,523,362,439), Ningmitai capsule combined with antibiotics (RR = 543271.32, 95%Cl: 5.69 ~ 26,528,137,117,330,244), Shuangdong capsule combined with antibiotics (RR = 282515.39, 95%Cl:2.66 ~ 14,302,174,706,164,270), Sanjin tablet (RR = 69283.44, 95% CI:1.07 ~ 3,113,089,480,585,731), Sanjin tablet combined with antibiotics (RR = 69283.44, 95% CI:1.07 ~ 3,113,089,480,585,731). The effective rate associated with the Ningmitai capsule was higher than that with the Sanjin tablet (RR = 162160.34, 95% CI:1.91 ~ 7,677,018,260,846,456) and the Yinhua Miyanling tablet combined with antibiotics (RR = 138134.37, 95% CI:1.44 ~ 6,915,099,000,393,685). The top 3 SUCRA rankings are: Xueniaoan capsule combined with antibiotics (85.57%), Ningmitai capsule combined with antibiotics (79.63%), Shuangdong capsule combined with antibiotics (66.49%). Adverse reactions included gastrointestinal reactions such as nausea, vomiting, diarrhea, and gastrointestinal discomfort, dizziness; and rash, as well as mild hepatic and renal impairment. The specific results are shown in Figure 4d and Table 5.

**Table 5:** League table (adverse events).

	Ab	BXFQ_Ab	FFSW	NMT	NMT_Ab	NTKKNQ	SD_Ab	SJP	SJP_Ab	XNA_Ab	YHMYL_Ab
Ab	Ab	0.99 (0.15, 6.17)	**149603.76 (1.86, 7,654,678,491,827,824)**	3140.11 (0, 1,383,056,115,013,177)	0.29 (0.05, 1.41)	410.83 (0, 1,597,639,504,046,406)	0.56 (0.08, 3.44)	2.16 (0.43, 13.03)	0.97 (0.14, 6.76)	0 (0, 2178.12)	1.11 (0.25, 4.87)
BXFQ_Ab	1.01 (0.16, 6.48)	BXFQ_Ab	**163612.61 (1.53, 7,149,846,523,362,439)**	3242.3 (0, 1,437,301,317,619,522)	0.29 (0.02, 3.36)	427.99 (0, 1,875,879,632,270,601)	0.56 (0.04, 7.57)	2.21 (0.19, 27.82)	0.98 (0.07, 13.73)	0 (0, 2688.21)	1.12 (0.11, 12.16)
FFSW	**0 (0, 0.54)**	**0 (0, 0.66)**	FFSW	0.01 (0, 144190316231.55)	**0 (0, 0.18)**	0 (0, 114322041263.24)	**0 (0, 0.38)**	**0 (0, 0.93)**	**0 (0, 0.52)**	0 (0, 1.04)	0 (0, 0.69)
NMT	0 (0, 81847.06)	0 (0, 91069.39)	101.7 (0, 1,455,791,560,336,344)	NMT	0 (0, 21943.73)	0.11 (0, 56,534,461,512,642)	0 (0, 53328.28)	0 (0, 168647.82)	0 (0, 81333.75)	0 (0, 8916.97)	0 (0, 97435.91)
NMT_Ab	3.45 (0.71, 19.95)	3.47 (0.3, 42.05)	**543271.32 (5.69, 26,528,137,117,330,244)**	11416.5 (0, 5,087,701,076,843,598)	NMT_Ab	1467.57 (0, 5,774,765,176,725,680)	1.96 (0.15, 23.78)	7.7 (0.78, 89.32)	3.39 (0.27, 46.35)	0 (0, 8113.96)	3.89 (0.44, 37.19)
NTKKNQ	0 (0, 7999371.82)	0 (0, 8539776.74)	796.83 (0, 61,029,698,029,209,688)	9.2 (0, 7,667,968,715,370,788)	0 (0, 2456635.24)	NTKKNQ	0 (0, 5017660.89)	0.01 (0, 17259296.43)	0 (0, 7946913.58)	0 (0, 507772.6)	0 (0, 9735260.4)
SD_Ab	1.8 (0.29, 12.82)	1.79 (0.13, 26.13)	**282515.39 (2.66, 14,302,174,706,164,270)**	5982.65 (0, 2,875,290,298,316,604)	0.51 (0.04, 6.58)	750.47 (0, 3,165,619,592,760,391)	SD_Ab	3.91 (0.35, 53.3)	1.75 (0.12, 27.18)	0 (0, 4718.36)	2.01 (0.19, 22.49)
SJP	0.46 (0.08, 2.33)	0.45 (0.04, 5.28)	**69283.44 (1.07, 3,113,089,480,585,731)**	1437.68 (0, 550,214,205,651,811)	0.13 (0.01, 1.28)	186.69 (0, 733,728,451,245,144)	0.26 (0.02, 2.86)	SJP	0.45 (0.07, 2.25)	0 (0, 1152.94)	0.51 (0.05, 4.49)
SJP_Ab	1.03 (0.15, 7.22)	1.02 (0.07, 14.57)	**162160.34 (1.91, 7,677,018,260,846,456)**	3365.79 (0, 1,388,296,658,157,482)	0.29 (0.02, 3.68)	430.68 (0, 1,882,149,350,508,364)	0.57 (0.04, 8.18)	2.21 (0.45, 13.9)	SJP_Ab	0 (0, 2835.17)	1.15 (0.1, 12.87)
XNA_Ab	13766.52 (0, 11,691,247,993,711,690)	14084.46 (0, 11,484,493,405,965,954)	18481271246.94 (0.96, 2.45886014710887e+24)	191709427.49 (0, 4.25319217920258e+24)	3848.79 (0, 3,353,593,683,749,319)	21329166.01 (0, 7.68568284181846e+23)	7686.91 (0, 7,429,780,721,896,445)	29711.5 (0, 24,921,489,719,378,708)	13209.71 (0, 11,985,754,689,092,606)	XNA_Ab	15844.44 (0, 13,342,995,079,553,980)
YHMYL_Ab	0.9 (0.21, 3.99)	0.89 (0.08, 9.39)	**138134.37 (1.44, 6,915,099,000,393,685)**	3006.46 (0, 1,227,694,191,496,793)	0.26 (0.03, 2.27)	366.33 (0, 1,515,610,936,684,963)	0.5 (0.04, 5.23)	1.96 (0.22, 19.36)	0.87 (0.08, 9.69)	0 (0, 1993.62)	YHMYL_Ab

Effect sizes where statistical differences exist are bolded.

### 3.5 Cluster analysis

**Figure 5:**
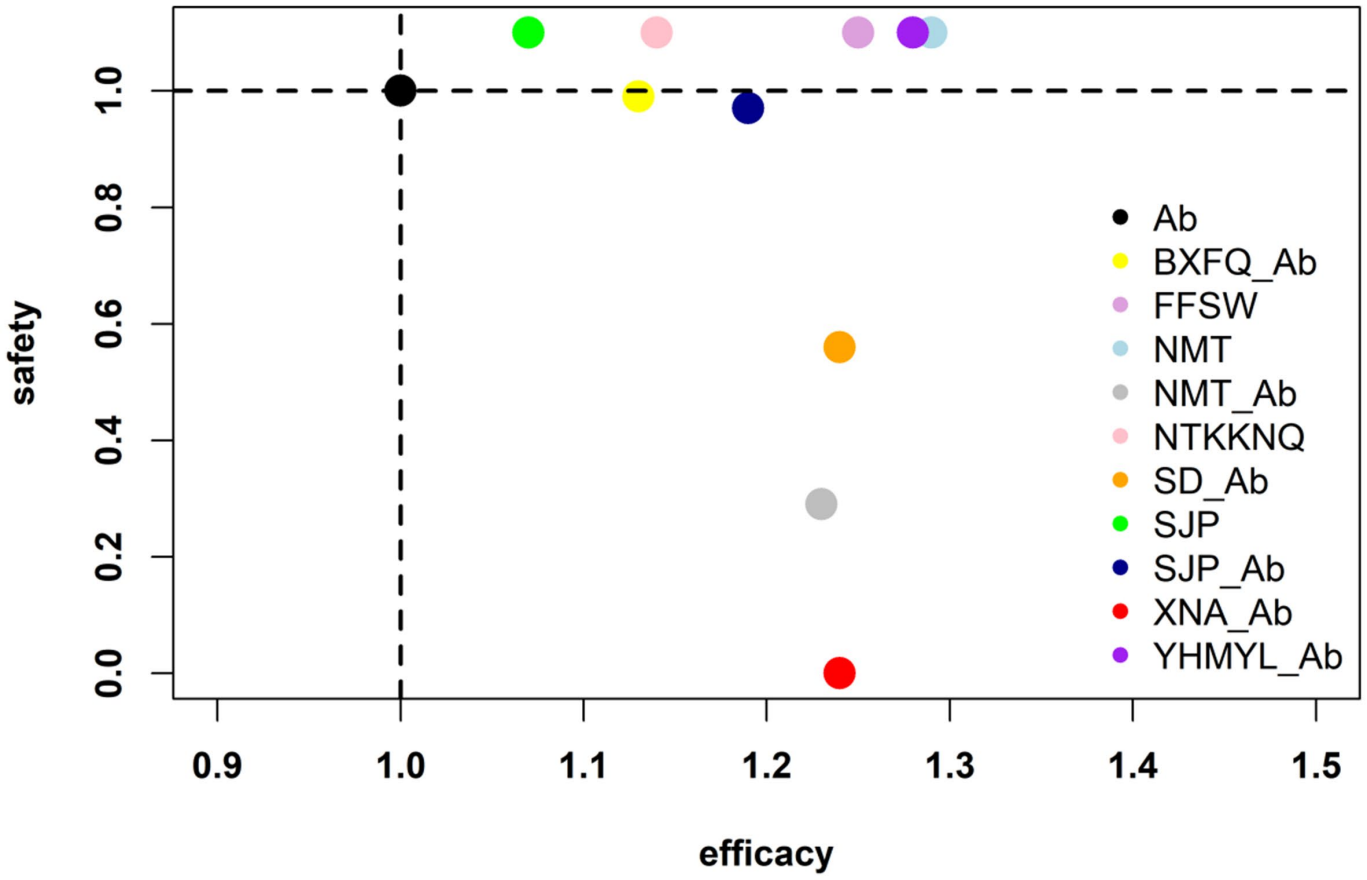
Interventions located in the lower right corner indicate the optimal combination therapy for the two different outcomes.

The impact of the intervention measures on two different outcomes was comprehensively compared through cluster analysis, including the total treatment effect and adverse effects of UTIs. The results are shown in Figure 5. Considering the combination of efficacy and safety, XNA_Ab may have good therapeutic efficacy and high safety.

### 3.6 Inconsistency test for closed loops

The node-splitting method was used for inconsistency testing. Inconsistent test results for cure rate, recurrence rate, and total effective rate were *p* > 0.05 (Supplementary Appendix D).

### 3.7 Measures of model fit

In the Bayesian model constructed in this study, the cure rate in the Brooks-Gelman-Rubin diagnosis plot during the iteration process reached a stable fusion of each Markov chain from the beginning, and the overlapping area accounted for most of the chain fluctuation range in the subsequent calculation. The naked eye could not identify the fluctuations of a single chain, and the degree of convergence was satisfactory. The density map is roughly normally distributed, the preset distribution gap is small, and the model convergence degree is satisfactory. The convergence diagnostic map shows the median value and 97.5% reduction factor after 10,000 pre-iterations, which quickly tends to 1 and reaches stability. The PSRF value is 1, indicating that the model convergence degree is satisfactory. The convergence evaluation results of the remaining outcome indicators showed that the model converged satisfactorily, the total number of iterations was sufficient, and the model fit was good; thus, the Bayesian NMA model constructed in this study could effectively evaluate the efficacy of different interventions in the treatment of UTI (Supplementary Appendix E).

### 3.8 Risk of bias

**Figure 6:**
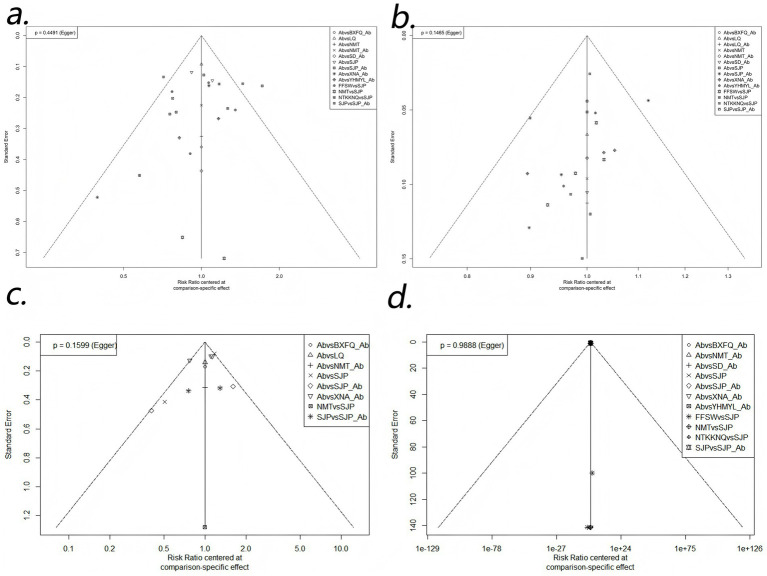
Comparison-correction funnel plot. **(a)** cure rate; **(b)** effective rate; **(c)** bacterial clearance; **(d)** adverse events.

In this NMA, there are multiple direct and indirect comparisons between drugs. A funnel plot for the publication bias test is shown in Figure 6. The dots in the funnel chart represent a one-to-one comparison between the two drugs, and the number of points represents the number of RCTs reported for comparison. If there was no significant effect of a small sample size or publication bias, the points in the funnel plot were symmetrical and evenly distributed on both sides of the vertical line. In the plot, the distribution of most points is relatively symmetrical and uniform, suggesting little evidence of publication bias. Egger’s test *p* > 0.05, suggesting no significant publication bias.

## 4 Discussion

We conducted a comprehensive search for relevant publications and pooled all available evidence from 23 RCTs involving 9 different Chinese medicines for the treatment of UTIs. We conducted a detailed comparison of the effectiveness and safety of the multiple pharmacological interventions. Considering the SUCRA results, NMT was most likely the best treatment option in terms of the cure rate, efficiency rate, and bacterial clearance. XNA_Ab was most likely the best treatment option in terms of adverse event rate. XNA_Ab is the best treatment option for UTI when combined with the overall efficiency ratio and adverse events. Clinicians should also choose the appropriate method according to the specific conditions of clinical patients.

UTIs are mostly caused by a single bacterial infection, with the main pathogen being gram-negative bacilli, and a few are fungi or gram-positive bacilli. Current guidelines recommend antibiotics such as fosfomycin, trometamol, pivmecillinam, or nitrofurantoin as the first-line treatment for UTIs. Clinically, broad-spectrum antibacterial drugs are mainly used, but with the widespread use of antibacterial drugs, the pathogenic bacteria of UTIs often develop resistance to commonly used antibacterial drugs, resulting in repeated symptoms and lingering conditions. In previous studies, Ningmitai capsules combined with antibiotic therapy demonstrated superiority in symptom resolution and normalization of laboratory indicators compared to antibiotic treatment alone (45). Sanjin tablets combined with antibiotics for acute lower urinary tract infections also showed advantages (46). Compared to antibiotics or Sanjin tablets alone, Compound Shiwei tablets are more effective in improving clinical efficiency and cure rates (47). Modern pharmacological research has shown that 27 of 138 known compounds in the Sanjin tablet have antibacterial and anti-inflammatory activities (17). Yinhua Miyanling tablets can change the growth cycle of bacteria, antibacterial endotoxin, inhibit NLRP3/caspase-1 inflammasomes, intervene in the renin-angiotensin-aldosterone system, and have bacteriostatic, anti-inflammatory, diuretic, and other effects (48). The ingredients in Ningmitai capsules inhibit the adhesion ability of bacteria such as Streptococcus, Enterococcus, Pseudomonas, Staphylococcus, and *Escherichia coli*, bacterial proliferation, and bacterial biofilm formation (49). The Shuangdong capsule demonstrates therapeutic efficacy against diabetes-associated UTIs. Its mechanism of action likely involves activation of the IRS1-PI3K-Akt signaling pathway, leading to elevated CXCL2 and MPO levels, enhanced innate immunity, and improved bacterial clearance (50). The present study involves a retrospective meta-analysis utilizing a Bayesian-based approach to assess the efficacy and safety of various CPMs and to rank their respective strengths and weaknesses.

Most clinical practice guidelines and research protocols classify UTIs as either uncomplicated or complicated infections based on the presence of comorbid conditions. An uncomplicated UTI is a localized urinary tract infection (cystitis) without any signs of systemic infection in either sex. Risk factors predisposing patients to a severe clinical course or treatment failure may be present and should be considered and addressed. A complicated UTI is a systemic urinary tract infection with or without localized symptoms originating from any site in the urinary tract in either sex. Risk factors predisposing to a severe clinical course or treatment failure may be present and should be considered and addressed (51). Lower urinary tract infections (UTIs) involve the urethra and bladder, whereas upper UTIs affect the kidneys and ureters. (52). Based on epidemiological characteristics, UTIs can be categorized as either community-acquired UTIs (CAUTIs) or healthcare-associated UTIs (HAUTIs) (53). The varying definitions of UTIs across different studies pose significant challenges in interpreting results and developing evidence-based guidelines (54). The new EAU classification categorizes UTIs as either localized or systemic according to the presence of specific clinical signs and symptoms: 1. Localized UTI: Cystitis without any signs or symptoms of systemic infection in either sex. 2. Systemic UTI: UTI with signs and symptoms of systemic infection, with or without localized symptoms originating from any site in the urinary tract in either sex (55). The studies included in this analysis did not provide an explicit classification of UTIs. Due to inconsistent classification criteria across studies and incomplete reporting of UTI categories in patient populations, future research should adopt more approaches based on the current UTI classification guidelines.

The selection of appropriate antimicrobial therapy based on the pathogen spectrum and susceptibility patterns remains the cornerstone of UTI management. For asymptomatic bacteriuria (ABU) with low-risk factors and uncomplicated cystitis with mild-to-moderate symptoms, observational management or symptomatic treatment alone may be considered without routine antimicrobial intervention (1). Several non-antimicrobial prophylactic options can be considered for patients with recurrent urinary tract infections, each with varying degrees of recommendation: hormonal replacement, immunoactive prophylaxis, probiotics, cranberry, D-mannose, intravesical instillation, and methenamine hippurate (56). It should be noted that current guidelines classify the evidence supporting these therapeutic approaches as low-grade, underscoring the need for additional clinical trials in the future. Natural medicines demonstrate fewer adverse side effects while offering superior therapeutic benefits. Currently, the acceptance of Chinese Patent Medicines (CPMs) remains limited in China and other countries/regions due to multiple factors. Primarily, the scarcity of robust clinical research evidence results in the uncertain therapeutic efficacy of CPMs (57). Furthermore, the application of CPMs must strictly follow the TCM principles of syndrome differentiation-based medication and individualized dosing protocols, which have not been fully comprehended by Western medical practitioners. Third, the dose-efficacy relationship of CPMs remains unclear. Finally, adverse effects frequently arise from contamination and improper usage, rather than inherent risks of the herbal components themselves, resulting in significant uncertainties regarding the safety and reliability profile of CPMs (58). Over the past three decades, evidence-based Chinese medicine (EBCM) has progressively emerged, evolving from initial conflicts between evidence-based medicine (EBM) and traditional Chinese medicine (TCM) practices toward their gradual integration (59).

Our studies were limited by the following: (1) most studies did not report the method of randomization, and none of the studies reported the study protocol, blinding, and concealment of the randomization protocol; (2) oly one study performed intention-to-intention analysis; (3) subgroup analyses of major risk factors such as age and sex were not performed in this review due to unclear classification and data standardization among all included RCTs, which had a significant impact on the development of UTI; (4) in various studies, the detection efficiency was low due to the small sample size and unstable efficacy indicators; and (5) indirect comparisons and imprecision (wide CIs or small sample sizes) further reduced confidence in the NMA estimates. The overall methodological quality of the included studies was found to be low. These studies involved a large number of CPMs, and we did not conduct specific TCM dialectical classification discussions. Given the limitations of this study, these conclusions should be validated by multicenter, high-quality, large-sample, randomized controlled trials.

## 5 Conclusion

Despite the many limitations of the current study, our NMA can still provide reasonable recommendations for the clinical use of CPMs. XNA_Ab emerged as a promising CPM candidate, demonstrating a favorable balance between efficacy and safety in our analysis. Given the multicomponent nature of CPMS, the use of CPM may vary depending on the patient, TCM syndrome, degree of involvement, severity, and symptoms. Therefore, doctors should consider individual characteristics, severity assessment, involvement area, and TCM theory when choosing. Further research should focus on mechanistic studies to elucidate XNA_Ab’s active components, dose-optimization trials for high-potential CPMs, and standardized protocols for syndrome-based CPM selection.

## Data Availability

The original contributions presented in the study are included in the article/Supplementary material, further inquiries can be directed to the corresponding author.
