# Supplementary material for: Efficacy and safety of Chinese patent medicine for urinary tract infections: a systematic review and network meta-analysis
Source: Front Med (Lausanne). 2025 Aug 21;12:1622999. doi: 10.3389/fmed.2025.1622999 (PMC12408631; doi:10.3389/fmed.2025.1622999)
Supplement: Supplementary file 1 [file Data_Sheet_1.docx]

Appendix A.

Pubmed：

1#((((((("Medicine, Chinese Traditional"[Mesh]) OR (Traditional Chinese Medicine)) OR (Chung I Hsueh)) OR (Hsueh, Chung I)) OR (Traditional Medicine, Chinese)) OR (Zhong Yi Xue)) OR (Chinese Traditional Medicine)) OR (Chinese Medicine, Traditional)

2#"urinary tract infections"[MeSH Terms] OR infection urinary tract OR infections urinary tract OR tract infections urinary OR urinary tract infection

1# AND 2#

Embase：

1#'urinary tract infection'/exp OR 'bacteriuria' OR 'urinary tract infection' OR '(urin* adj3 infection*)' OR 'bladder infection' OR 'bladder infection'

2#'Chinese medicine'/exp OR 'Chinese drug'

1# AND 2#

Ovid medline：

1# exp Urinary Tract Infections/

2# Infection, Urinary Tract.mp.

3# exp Bacteriuria/

4# 1# OR 2# OR 3#

5# Medicine, Chinese Traditional/ or Drugs, Chinese Herbal/ or Chinese medicine.mp. or exp Medicine, East Asian Traditional/

4# AND 5#

Cnki：

中成药 + 传统中成药 + 中医治疗 + 中药 + 中医疗法

泌尿系感染 + 尿道炎 + 尿路感染

临床研究 + 随机对照 + 疗效观察

Wangfang data：

尿道炎 OR 尿路感染 OR 下尿路感染 OR 泌尿系感染

中成药 OR 传统中成药 OR 中医治疗 OR 中药 OR 中医疗法 OR 中草药

随机对照 OR 临床研究

Appendix B. GRADE quality assessment

Appendix B.1. GRADE quality assessment for cure rate

| Comparison | Number of studies | Within-study bias | Reporting bias | Indirectness | Imprecision | Heterogeneity | Incoherence | Confidence rating | Reason(s) for downgrading |
| --- | --- | --- | --- | --- | --- | --- | --- | --- | --- |
| Ab:BXFQ_Ab | 1 | Some concerns | Low risk | No concerns | Major concerns | No concerns | No concerns | Very low | ["Within-study bias","Imprecision"] |
| Ab:LQ | 1 | Some concerns | Low risk | No concerns | Major concerns | No concerns | No concerns | Very low | ["Within-study bias","Imprecision"] |
| Ab:NMT | 1 | Some concerns | Low risk | No concerns | No concerns | No concerns | No concerns | Moderate | ["Within-study bias"] |
| Ab:NMT_Ab | 1 | Some concerns | Low risk | No concerns | Major concerns | No concerns | No concerns | Very low | ["Within-study bias","Imprecision"] |
| Ab:SD_Ab | 1 | Some concerns | Low risk | No concerns | Major concerns | No concerns | No concerns | Very low | ["Within-study bias","Imprecision"] |
| Ab:SJP | 2 | No concerns | Low risk | No concerns | Major concerns | No concerns | No concerns | Low | ["Imprecision"] |
| Ab:SJP_Ab | 5 | No concerns | Low risk | No concerns | Major concerns | No concerns | No concerns | Low | ["Imprecision"] |
| Ab:XNA_Ab | 3 | Some concerns | Low risk | No concerns | No concerns | Major concerns | No concerns | Very low | ["Within-study bias","Heterogeneity"] |
| Ab:YHMYL_Ab | 2 | Some concerns | Low risk | No concerns | Major concerns | No concerns | No concerns | Very low | ["Within-study bias","Imprecision"] |
| FFSW:SJP | 4 | Some concerns | Low risk | No concerns | No concerns | Major concerns | No concerns | Very low | ["Within-study bias","Heterogeneity"] |
| NMT:SJP | 2 | Some concerns | Low risk | No concerns | No concerns | No concerns | No concerns | Moderate | ["Within-study bias"] |
| NTKKNQ:SJP | 2 | Major concerns | Low risk | No concerns | No concerns | Major concerns | No concerns | Very low | ["Within-study bias","Heterogeneity"] |
| SJP:SJP_Ab | 2 | No concerns | Low risk | No concerns | Major concerns | No concerns | No concerns | Low | ["Imprecision"] |
| Ab:FFSW | 0 | Some concerns | Low risk | No concerns | Major concerns | No concerns | No concerns | Very low | ["Within-study bias","Imprecision"] |
| Ab:NTKKNQ | 0 | Some concerns | Low risk | No concerns | No concerns | Major concerns | No concerns | Very low | ["Within-study bias","Heterogeneity"] |
| BXFQ_Ab:FFSW | 0 | Some concerns | Low risk | No concerns | Major concerns | No concerns | No concerns | Very low | ["Within-study bias","Imprecision"] |
| BXFQ_Ab:LQ | 0 | Some concerns | Low risk | No concerns | Major concerns | No concerns | No concerns | Very low | ["Within-study bias","Imprecision"] |
| BXFQ_Ab:NMT | 0 | Some concerns | Low risk | No concerns | Major concerns | No concerns | No concerns | Very low | ["Within-study bias","Imprecision"] |
| BXFQ_Ab:NMT_Ab | 0 | Some concerns | Low risk | No concerns | Major concerns | No concerns | No concerns | Very low | ["Within-study bias","Imprecision"] |
| BXFQ_Ab:NTKKNQ | 0 | Some concerns | Low risk | No concerns | Major concerns | No concerns | No concerns | Very low | ["Within-study bias","Imprecision"] |
| BXFQ_Ab:SD_Ab | 0 | Some concerns | Low risk | No concerns | Major concerns | No concerns | No concerns | Very low | ["Within-study bias","Imprecision"] |
| BXFQ_Ab:SJP | 0 | Some concerns | Low risk | No concerns | Major concerns | No concerns | No concerns | Very low | ["Within-study bias","Imprecision"] |
| BXFQ_Ab:SJP_Ab | 0 | Some concerns | Low risk | No concerns | Major concerns | No concerns | No concerns | Very low | ["Within-study bias","Imprecision"] |
| BXFQ_Ab:XNA_Ab | 0 | Some concerns | Low risk | No concerns | Major concerns | No concerns | No concerns | Very low | ["Within-study bias","Imprecision"] |
| BXFQ_Ab:YHMYL_Ab | 0 | Some concerns | Low risk | No concerns | Major concerns | No concerns | No concerns | Very low | ["Within-study bias","Imprecision"] |
| FFSW:LQ | 0 | Some concerns | Low risk | No concerns | Major concerns | No concerns | No concerns | Very low | ["Within-study bias","Imprecision"] |
| FFSW:NMT | 0 | Some concerns | Low risk | No concerns | Major concerns | No concerns | No concerns | Very low | ["Within-study bias","Imprecision"] |
| FFSW:NMT_Ab | 0 | Some concerns | Low risk | No concerns | Major concerns | No concerns | No concerns | Very low | ["Within-study bias","Imprecision"] |
| FFSW:NTKKNQ | 0 | Some concerns | Low risk | No concerns | Major concerns | No concerns | No concerns | Very low | ["Within-study bias","Imprecision"] |
| FFSW:SD_Ab | 0 | Some concerns | Low risk | No concerns | Major concerns | No concerns | No concerns | Very low | ["Within-study bias","Imprecision"] |
| FFSW:SJP_Ab | 0 | Some concerns | Low risk | No concerns | Major concerns | No concerns | No concerns | Very low | ["Within-study bias","Imprecision"] |
| FFSW:XNA_Ab | 0 | Some concerns | Low risk | No concerns | Major concerns | No concerns | No concerns | Very low | ["Within-study bias","Imprecision"] |
| FFSW:YHMYL_Ab | 0 | Some concerns | Low risk | No concerns | Major concerns | No concerns | No concerns | Very low | ["Within-study bias","Imprecision"] |
| LQ:NMT | 0 | Some concerns | Low risk | No concerns | No concerns | No concerns | No concerns | Moderate | ["Within-study bias"] |
| LQ:NMT_Ab | 0 | Some concerns | Low risk | No concerns | Major concerns | No concerns | No concerns | Very low | ["Within-study bias","Imprecision"] |
| LQ:NTKKNQ | 0 | Some concerns | Low risk | No concerns | Major concerns | No concerns | No concerns | Very low | ["Within-study bias","Imprecision"] |
| LQ:SD_Ab | 0 | Some concerns | Low risk | No concerns | Major concerns | No concerns | No concerns | Very low | ["Within-study bias","Imprecision"] |
| LQ:SJP | 0 | Some concerns | Low risk | No concerns | Major concerns | No concerns | No concerns | Very low | ["Within-study bias","Imprecision"] |
| LQ:SJP_Ab | 0 | Some concerns | Low risk | No concerns | Major concerns | No concerns | No concerns | Very low | ["Within-study bias","Imprecision"] |
| LQ:XNA_Ab | 0 | Some concerns | Low risk | No concerns | Major concerns | No concerns | No concerns | Very low | ["Within-study bias","Imprecision"] |
| LQ:YHMYL_Ab | 0 | Some concerns | Low risk | No concerns | Major concerns | No concerns | No concerns | Very low | ["Within-study bias","Imprecision"] |
| NMT:NMT_Ab | 0 | Some concerns | Low risk | No concerns | Major concerns | No concerns | No concerns | Very low | ["Within-study bias","Imprecision"] |
| NMT:NTKKNQ | 0 | Some concerns | Low risk | No concerns | Major concerns | No concerns | No concerns | Very low | ["Within-study bias","Imprecision"] |
| NMT:SD_Ab | 0 | Some concerns | Low risk | No concerns | Major concerns | No concerns | No concerns | Very low | ["Within-study bias","Imprecision"] |
| NMT:SJP_Ab | 0 | Some concerns | Low risk | No concerns | No concerns | No concerns | No concerns | Moderate | ["Within-study bias"] |
| NMT:XNA_Ab | 0 | Some concerns | Low risk | No concerns | Major concerns | No concerns | No concerns | Very low | ["Within-study bias","Imprecision"] |
| NMT:YHMYL_Ab | 0 | Some concerns | Low risk | No concerns | No concerns | Major concerns | No concerns | Very low | ["Within-study bias","Heterogeneity"] |
| NMT_Ab:NTKKNQ | 0 | Some concerns | Low risk | No concerns | Major concerns | No concerns | No concerns | Very low | ["Within-study bias","Imprecision"] |
| NMT_Ab:SD_Ab | 0 | Some concerns | Low risk | No concerns | Major concerns | No concerns | No concerns | Very low | ["Within-study bias","Imprecision"] |
| NMT_Ab:SJP | 0 | Some concerns | Low risk | No concerns | Major concerns | No concerns | No concerns | Very low | ["Within-study bias","Imprecision"] |
| NMT_Ab:SJP_Ab | 0 | Some concerns | Low risk | No concerns | Major concerns | No concerns | No concerns | Very low | ["Within-study bias","Imprecision"] |
| NMT_Ab:XNA_Ab | 0 | Some concerns | Low risk | No concerns | Major concerns | No concerns | No concerns | Very low | ["Within-study bias","Imprecision"] |
| NMT_Ab:YHMYL_Ab | 0 | Some concerns | Low risk | No concerns | Major concerns | No concerns | No concerns | Very low | ["Within-study bias","Imprecision"] |
| NTKKNQ:SD_Ab | 0 | Some concerns | Low risk | No concerns | Major concerns | No concerns | No concerns | Very low | ["Within-study bias","Imprecision"] |
| NTKKNQ:SJP_Ab | 0 | Some concerns | Low risk | No concerns | Major concerns | No concerns | No concerns | Very low | ["Within-study bias","Imprecision"] |
| NTKKNQ:XNA_Ab | 0 | Some concerns | Low risk | No concerns | Major concerns | No concerns | No concerns | Very low | ["Within-study bias","Imprecision"] |
| NTKKNQ:YHMYL_Ab | 0 | Some concerns | Low risk | No concerns | Major concerns | No concerns | No concerns | Very low | ["Within-study bias","Imprecision"] |
| SD_Ab:SJP | 0 | Some concerns | Low risk | No concerns | Major concerns | No concerns | No concerns | Very low | ["Within-study bias","Imprecision"] |
| SD_Ab:SJP_Ab | 0 | Some concerns | Low risk | No concerns | Major concerns | No concerns | No concerns | Very low | ["Within-study bias","Imprecision"] |
| SD_Ab:XNA_Ab | 0 | Some concerns | Low risk | No concerns | Major concerns | No concerns | No concerns | Very low | ["Within-study bias","Imprecision"] |
| SD_Ab:YHMYL_Ab | 0 | Some concerns | Low risk | No concerns | Major concerns | No concerns | No concerns | Very low | ["Within-study bias","Imprecision"] |
| SJP:XNA_Ab | 0 | Some concerns | Low risk | No concerns | Major concerns | No concerns | No concerns | Very low | ["Within-study bias","Imprecision"] |
| SJP:YHMYL_Ab | 0 | Some concerns | Low risk | No concerns | Major concerns | No concerns | No concerns | Very low | ["Within-study bias","Imprecision"] |
| SJP_Ab:XNA_Ab | 0 | Some concerns | Low risk | No concerns | Major concerns | No concerns | No concerns | Very low | ["Within-study bias","Imprecision"] |
| SJP_Ab:YHMYL_Ab | 0 | Some concerns | Low risk | No concerns | Major concerns | No concerns | No concerns | Very low | ["Within-study bias","Imprecision"] |
| XNA_Ab:YHMYL_Ab | 0 | Some concerns | Low risk | No concerns | Major concerns | No concerns | No concerns | Very low | ["Within-study bias","Imprecision"] |

Appendix B.2. GRADE quality assessment for effective rate

| Comparison | Number of studies | Within-study bias | Reporting bias | Indirectness | Imprecision | Heterogeneity | Incoherence | Confidence rating | Reason(s) for downgrading |
| --- | --- | --- | --- | --- | --- | --- | --- | --- | --- |
| Ab:BXFQ_Ab | 1 | Some concerns | Low risk | No concerns | Major concerns | No concerns | Major concerns | Very low | ["Within-study bias","Imprecision"] |
| Ab:LQ | 1 | Some concerns | Low risk | No concerns | No concerns | No concerns | Major concerns | Moderate | ["Within-study bias"] |
| Ab:LQ_Ab | 1 | Some concerns | Low risk | No concerns | No concerns | Major concerns | Major concerns | Very low | ["Within-study bias","Heterogeneity"] |
| Ab:NMT | 1 | Some concerns | Low risk | No concerns | No concerns | No concerns | No concerns | Moderate | ["Within-study bias"] |
| Ab:NMT_Ab | 1 | Some concerns | Low risk | No concerns | No concerns | Major concerns | Major concerns | Very low | ["Within-study bias","Heterogeneity"] |
| Ab:SD_Ab | 1 | Some concerns | Low risk | No concerns | Major concerns | No concerns | Major concerns | Very low | ["Within-study bias","Imprecision"] |
| Ab:SJP | 1 | No concerns | Low risk | No concerns | Major concerns | No concerns | Major concerns | Low | ["Imprecision"] |
| Ab:SJP_Ab | 4 | Some concerns | Low risk | No concerns | No concerns | No concerns | No concerns | Moderate | ["Within-study bias"] |
| Ab:XNA_Ab | 3 | Some concerns | Low risk | No concerns | No concerns | No concerns | Major concerns | Moderate | ["Within-study bias"] |
| Ab:YHMYL_Ab | 2 | Some concerns | Low risk | No concerns | No concerns | No concerns | Major concerns | Moderate | ["Within-study bias"] |
| FFSW:SJP | 4 | Some concerns | Low risk | No concerns | No concerns | No concerns | Major concerns | Moderate | ["Within-study bias"] |
| NMT:SJP | 2 | Some concerns | Low risk | No concerns | No concerns | No concerns | No concerns | Moderate | ["Within-study bias"] |
| NTKKNQ:SJP | 2 | Major concerns | Low risk | No concerns | Major concerns | No concerns | Major concerns | Very low | ["Within-study bias","Imprecision"] |
| SJP:SJP_Ab | 1 | No concerns | Low risk | No concerns | Major concerns | No concerns | Major concerns | Low | ["Imprecision"] |
| Ab:FFSW | 0 | Some concerns | Low risk | No concerns | No concerns | No concerns | Major concerns | Moderate | ["Within-study bias"] |
| Ab:NTKKNQ | 0 | Some concerns | Low risk | No concerns | Major concerns | No concerns | Major concerns | Very low | ["Within-study bias","Imprecision"] |
| BXFQ_Ab:FFSW | 0 | Some concerns | Low risk | No concerns | Major concerns | No concerns | Major concerns | Very low | ["Within-study bias","Imprecision"] |
| BXFQ_Ab:LQ | 0 | Some concerns | Low risk | No concerns | Major concerns | No concerns | Major concerns | Very low | ["Within-study bias","Imprecision"] |
| BXFQ_Ab:LQ_Ab | 0 | Some concerns | Low risk | No concerns | Major concerns | No concerns | Major concerns | Very low | ["Within-study bias","Imprecision"] |
| BXFQ_Ab:NMT | 0 | Some concerns | Low risk | No concerns | Major concerns | No concerns | Major concerns | Very low | ["Within-study bias","Imprecision"] |
| BXFQ_Ab:NMT_Ab | 0 | Some concerns | Low risk | No concerns | Major concerns | No concerns | Major concerns | Very low | ["Within-study bias","Imprecision"] |
| BXFQ_Ab:NTKKNQ | 0 | Some concerns | Low risk | No concerns | Major concerns | No concerns | Major concerns | Very low | ["Within-study bias","Imprecision"] |
| BXFQ_Ab:SD_Ab | 0 | Some concerns | Low risk | No concerns | Major concerns | No concerns | Major concerns | Very low | ["Within-study bias","Imprecision"] |
| BXFQ_Ab:SJP | 0 | Some concerns | Low risk | No concerns | Major concerns | No concerns | Major concerns | Very low | ["Within-study bias","Imprecision"] |
| BXFQ_Ab:SJP_Ab | 0 | Some concerns | Low risk | No concerns | Major concerns | No concerns | Major concerns | Very low | ["Within-study bias","Imprecision"] |
| BXFQ_Ab:XNA_Ab | 0 | Some concerns | Low risk | No concerns | Major concerns | No concerns | Major concerns | Very low | ["Within-study bias","Imprecision"] |
| BXFQ_Ab:YHMYL_Ab | 0 | Some concerns | Low risk | No concerns | Major concerns | No concerns | Major concerns | Very low | ["Within-study bias","Imprecision"] |
| FFSW:LQ | 0 | Some concerns | Low risk | No concerns | Major concerns | No concerns | Major concerns | Very low | ["Within-study bias","Imprecision"] |
| FFSW:LQ_Ab | 0 | Some concerns | Low risk | No concerns | Major concerns | No concerns | Major concerns | Very low | ["Within-study bias","Imprecision"] |
| FFSW:NMT | 0 | Some concerns | Low risk | No concerns | Major concerns | No concerns | Major concerns | Very low | ["Within-study bias","Imprecision"] |
| FFSW:NMT_Ab | 0 | Some concerns | Low risk | No concerns | Major concerns | No concerns | Major concerns | Very low | ["Within-study bias","Imprecision"] |
| FFSW:NTKKNQ | 0 | Some concerns | Low risk | No concerns | Major concerns | No concerns | Major concerns | Very low | ["Within-study bias","Imprecision"] |
| FFSW:SD_Ab | 0 | Some concerns | Low risk | No concerns | Major concerns | No concerns | Major concerns | Very low | ["Within-study bias","Imprecision"] |
| FFSW:SJP_Ab | 0 | Some concerns | Low risk | No concerns | Major concerns | No concerns | Major concerns | Very low | ["Within-study bias","Imprecision"] |
| FFSW:XNA_Ab | 0 | Some concerns | Low risk | No concerns | Major concerns | No concerns | Major concerns | Very low | ["Within-study bias","Imprecision"] |
| FFSW:YHMYL_Ab | 0 | Some concerns | Low risk | No concerns | Major concerns | No concerns | Major concerns | Very low | ["Within-study bias","Imprecision"] |
| LQ:LQ_Ab | 0 | Some concerns | Low risk | No concerns | Major concerns | No concerns | Major concerns | Very low | ["Within-study bias","Imprecision"] |
| LQ:NMT | 0 | Some concerns | Low risk | No concerns | Major concerns | No concerns | Major concerns | Very low | ["Within-study bias","Imprecision"] |
| LQ:NMT_Ab | 0 | Some concerns | Low risk | No concerns | Major concerns | No concerns | Major concerns | Very low | ["Within-study bias","Imprecision"] |
| LQ:NTKKNQ | 0 | Some concerns | Low risk | No concerns | Major concerns | No concerns | Major concerns | Very low | ["Within-study bias","Imprecision"] |
| LQ:SD_Ab | 0 | Some concerns | Low risk | No concerns | Major concerns | No concerns | Major concerns | Very low | ["Within-study bias","Imprecision"] |
| LQ:SJP | 0 | Some concerns | Low risk | No concerns | Major concerns | No concerns | Major concerns | Very low | ["Within-study bias","Imprecision"] |
| LQ:SJP_Ab | 0 | Some concerns | Low risk | No concerns | Major concerns | No concerns | Major concerns | Very low | ["Within-study bias","Imprecision"] |
| LQ:XNA_Ab | 0 | Some concerns | Low risk | No concerns | Major concerns | No concerns | Major concerns | Very low | ["Within-study bias","Imprecision"] |
| LQ:YHMYL_Ab | 0 | Some concerns | Low risk | No concerns | Major concerns | No concerns | Major concerns | Very low | ["Within-study bias","Imprecision"] |
| LQ_Ab:NMT | 0 | Some concerns | Low risk | No concerns | Major concerns | No concerns | Major concerns | Very low | ["Within-study bias","Imprecision"] |
| LQ_Ab:NMT_Ab | 0 | Some concerns | Low risk | No concerns | Major concerns | No concerns | Major concerns | Very low | ["Within-study bias","Imprecision"] |
| LQ_Ab:NTKKNQ | 0 | Some concerns | Low risk | No concerns | Major concerns | No concerns | Major concerns | Very low | ["Within-study bias","Imprecision"] |
| LQ_Ab:SD_Ab | 0 | Some concerns | Low risk | No concerns | Major concerns | No concerns | Major concerns | Very low | ["Within-study bias","Imprecision"] |
| LQ_Ab:SJP | 0 | Some concerns | Low risk | No concerns | Major concerns | No concerns | Major concerns | Very low | ["Within-study bias","Imprecision"] |
| LQ_Ab:SJP_Ab | 0 | Some concerns | Low risk | No concerns | Major concerns | No concerns | Major concerns | Very low | ["Within-study bias","Imprecision"] |
| LQ_Ab:XNA_Ab | 0 | Some concerns | Low risk | No concerns | Major concerns | No concerns | Major concerns | Very low | ["Within-study bias","Imprecision"] |
| LQ_Ab:YHMYL_Ab | 0 | Some concerns | Low risk | No concerns | Major concerns | No concerns | Major concerns | Very low | ["Within-study bias","Imprecision"] |
| NMT:NMT_Ab | 0 | Some concerns | Low risk | No concerns | Major concerns | No concerns | Major concerns | Very low | ["Within-study bias","Imprecision"] |
| NMT:NTKKNQ | 0 | Some concerns | Low risk | No concerns | Major concerns | No concerns | Major concerns | Very low | ["Within-study bias","Imprecision"] |
| NMT:SD_Ab | 0 | Some concerns | Low risk | No concerns | Major concerns | No concerns | Major concerns | Very low | ["Within-study bias","Imprecision"] |
| NMT:SJP_Ab | 0 | Some concerns | Low risk | No concerns | Major concerns | No concerns | Major concerns | Very low | ["Within-study bias","Imprecision"] |
| NMT:XNA_Ab | 0 | Some concerns | Low risk | No concerns | Major concerns | No concerns | Major concerns | Very low | ["Within-study bias","Imprecision"] |
| NMT:YHMYL_Ab | 0 | Some concerns | Low risk | No concerns | Major concerns | No concerns | Major concerns | Very low | ["Within-study bias","Imprecision"] |
| NMT_Ab:NTKKNQ | 0 | Some concerns | Low risk | No concerns | Major concerns | No concerns | Major concerns | Very low | ["Within-study bias","Imprecision"] |
| NMT_Ab:SD_Ab | 0 | Some concerns | Low risk | No concerns | Major concerns | No concerns | Major concerns | Very low | ["Within-study bias","Imprecision"] |
| NMT_Ab:SJP | 0 | Some concerns | Low risk | No concerns | Major concerns | No concerns | Major concerns | Very low | ["Within-study bias","Imprecision"] |
| NMT_Ab:SJP_Ab | 0 | Some concerns | Low risk | No concerns | Major concerns | No concerns | Major concerns | Very low | ["Within-study bias","Imprecision"] |
| NMT_Ab:XNA_Ab | 0 | Some concerns | Low risk | No concerns | Major concerns | No concerns | Major concerns | Very low | ["Within-study bias","Imprecision"] |
| NMT_Ab:YHMYL_Ab | 0 | Some concerns | Low risk | No concerns | Major concerns | No concerns | Major concerns | Very low | ["Within-study bias","Imprecision"] |
| NTKKNQ:SD_Ab | 0 | Some concerns | Low risk | No concerns | Major concerns | No concerns | Major concerns | Very low | ["Within-study bias","Imprecision"] |
| NTKKNQ:SJP_Ab | 0 | Some concerns | Low risk | No concerns | Major concerns | No concerns | Major concerns | Very low | ["Within-study bias","Imprecision"] |
| NTKKNQ:XNA_Ab | 0 | Some concerns | Low risk | No concerns | Major concerns | No concerns | Major concerns | Very low | ["Within-study bias","Imprecision"] |
| NTKKNQ:YHMYL_Ab | 0 | Some concerns | Low risk | No concerns | Major concerns | No concerns | Major concerns | Very low | ["Within-study bias","Imprecision"] |
| SD_Ab:SJP | 0 | Some concerns | Low risk | No concerns | Major concerns | No concerns | Major concerns | Very low | ["Within-study bias","Imprecision"] |
| SD_Ab:SJP_Ab | 0 | Some concerns | Low risk | No concerns | Major concerns | No concerns | Major concerns | Very low | ["Within-study bias","Imprecision"] |
| SD_Ab:XNA_Ab | 0 | Some concerns | Low risk | No concerns | Major concerns | No concerns | Major concerns | Very low | ["Within-study bias","Imprecision"] |
| SD_Ab:YHMYL_Ab | 0 | Some concerns | Low risk | No concerns | Major concerns | No concerns | Major concerns | Very low | ["Within-study bias","Imprecision"] |
| SJP:XNA_Ab | 0 | Some concerns | Low risk | No concerns | No concerns | Major concerns | Major concerns | Very low | ["Within-study bias","Heterogeneity"] |
| SJP:YHMYL_Ab | 0 | Some concerns | Low risk | No concerns | Major concerns | No concerns | Major concerns | Very low | ["Within-study bias","Imprecision"] |
| SJP_Ab:XNA_Ab | 0 | Some concerns | Low risk | No concerns | Major concerns | No concerns | Major concerns | Very low | ["Within-study bias","Imprecision"] |
| SJP_Ab:YHMYL_Ab | 0 | Some concerns | Low risk | No concerns | Major concerns | No concerns | Major concerns | Very low | ["Within-study bias","Imprecision"] |
| XNA_Ab:YHMYL_Ab | 0 | Some concerns | Low risk | No concerns | Major concerns | No concerns | Major concerns | Very low | ["Within-study bias","Imprecision"] |

Appendix B.3. GRADE quality assessment for bacterial clearance

| Comparison | Number of studies | Within-study bias | Reporting bias | Indirectness | Imprecision | Heterogeneity | Incoherence | Confidence rating | Reason(s) for downgrading |
| --- | --- | --- | --- | --- | --- | --- | --- | --- | --- |
| Ab:BXFQ_Ab | 1 | Some concerns | Low risk | No concerns | Major concerns | No concerns | Major concerns | Very low | ["Within-study bias","Imprecision"] |
| Ab:LQ | 1 | Some concerns | Low risk | No concerns | Major concerns | No concerns | Major concerns | Very low | ["Within-study bias","Imprecision"] |
| Ab:NMT_Ab | 1 | Some concerns | Low risk | No concerns | Major concerns | No concerns | Major concerns | Very low | ["Within-study bias","Imprecision"] |
| Ab:SJP | 2 | No concerns | Low risk | No concerns | Major concerns | No concerns | No concerns | Low | ["Imprecision"] |
| Ab:SJP_Ab | 2 | No concerns | Low risk | No concerns | Major concerns | No concerns | No concerns | Low | ["Imprecision"] |
| Ab:XNA_Ab | 3 | Some concerns | Low risk | No concerns | No concerns | Major concerns | Major concerns | Very low | ["Within-study bias","Heterogeneity"] |
| NMT:SJP | 1 | Some concerns | Low risk | No concerns | Major concerns | No concerns | Major concerns | Very low | ["Within-study bias","Imprecision"] |
| SJP:SJP_Ab | 2 | No concerns | Low risk | No concerns | Major concerns | No concerns | No concerns | Low | ["Imprecision"] |
| Ab:NMT | 0 | No concerns | Low risk | No concerns | Major concerns | No concerns | Major concerns | Low | ["Imprecision"] |
| BXFQ_Ab:LQ | 0 | Some concerns | Low risk | No concerns | Major concerns | No concerns | Major concerns | Very low | ["Within-study bias","Imprecision"] |
| BXFQ_Ab:NMT | 0 | Some concerns | Low risk | No concerns | Major concerns | No concerns | Major concerns | Very low | ["Within-study bias","Imprecision"] |
| BXFQ_Ab:NMT_Ab | 0 | Some concerns | Low risk | No concerns | Major concerns | No concerns | Major concerns | Very low | ["Within-study bias","Imprecision"] |
| BXFQ_Ab:SJP | 0 | No concerns | Low risk | No concerns | Major concerns | No concerns | Major concerns | Low | ["Imprecision"] |
| BXFQ_Ab:SJP_Ab | 0 | No concerns | Low risk | No concerns | Major concerns | No concerns | Major concerns | Low | ["Imprecision"] |
| BXFQ_Ab:XNA_Ab | 0 | Some concerns | Low risk | No concerns | Major concerns | No concerns | Major concerns | Very low | ["Within-study bias","Imprecision"] |
| LQ:NMT | 0 | Some concerns | Low risk | No concerns | Major concerns | No concerns | Major concerns | Very low | ["Within-study bias","Imprecision"] |
| LQ:NMT_Ab | 0 | Some concerns | Low risk | No concerns | Major concerns | No concerns | Major concerns | Very low | ["Within-study bias","Imprecision"] |
| LQ:SJP | 0 | No concerns | Low risk | No concerns | Major concerns | No concerns | Major concerns | Low | ["Imprecision"] |
| LQ:SJP_Ab | 0 | No concerns | Low risk | No concerns | Major concerns | No concerns | Major concerns | Low | ["Imprecision"] |
| LQ:XNA_Ab | 0 | Some concerns | Low risk | No concerns | No concerns | Major concerns | Major concerns | Very low | ["Within-study bias","Heterogeneity"] |
| NMT:NMT_Ab | 0 | Some concerns | Low risk | No concerns | Major concerns | No concerns | Major concerns | Very low | ["Within-study bias","Imprecision"] |
| NMT:SJP_Ab | 0 | No concerns | Low risk | No concerns | Major concerns | No concerns | Major concerns | Low | ["Imprecision"] |
| NMT:XNA_Ab | 0 | Some concerns | Low risk | No concerns | Major concerns | No concerns | Major concerns | Very low | ["Within-study bias","Imprecision"] |
| NMT_Ab:SJP | 0 | No concerns | Low risk | No concerns | Major concerns | No concerns | Major concerns | Low | ["Imprecision"] |
| NMT_Ab:SJP_Ab | 0 | No concerns | Low risk | No concerns | Major concerns | No concerns | Major concerns | Low | ["Imprecision"] |
| NMT_Ab:XNA_Ab | 0 | Some concerns | Low risk | No concerns | Major concerns | No concerns | Major concerns | Very low | ["Within-study bias","Imprecision"] |
| SJP:XNA_Ab | 0 | No concerns | Low risk | No concerns | Major concerns | No concerns | Major concerns | Low | ["Imprecision"] |
| SJP_Ab:XNA_Ab | 0 | No concerns | Low risk | No concerns | No concerns | Major concerns | Major concerns | Low | ["Heterogeneity"] |

Appendix B.4. GRADE quality assessment for adverse events

| Comparison | Number of studies | Within-study bias | Reporting bias | Indirectness | Imprecision | Heterogeneity | Incoherence | Confidence rating | Reason(s) for downgrading |
| --- | --- | --- | --- | --- | --- | --- | --- | --- | --- |
| Ab:BXFQ_Ab | 1 | Some concerns | Low risk | No concerns | Major concerns | No concerns | No concerns | Very low | ["Within-study bias","Imprecision"] |
| Ab:NMT_Ab | 1 | Some concerns | Low risk | No concerns | No concerns | Major concerns | No concerns | Very low | ["Within-study bias","Heterogeneity"] |
| Ab:SD_Ab | 1 | Some concerns | Low risk | No concerns | Major concerns | No concerns | No concerns | Very low | ["Within-study bias","Imprecision"] |
| Ab:SJP | 2 | No concerns | Low risk | No concerns | Major concerns | No concerns | No concerns | Low | ["Imprecision"] |
| Ab:SJP_Ab | 3 | No concerns | Low risk | No concerns | Major concerns | No concerns | No concerns | Low | ["Imprecision"] |
| Ab:XNA_Ab | 3 | Some concerns | Low risk | No concerns | Major concerns | No concerns | No concerns | Very low | ["Within-study bias","Imprecision"] |
| Ab:YHMYL_Ab | 1 | Some concerns | Low risk | No concerns | Major concerns | No concerns | No concerns | Very low | ["Within-study bias","Imprecision"] |
| FFSW:SJP | 2 | Some concerns | Low risk | No concerns | Major concerns | No concerns | No concerns | Very low | ["Within-study bias","Imprecision"] |
| NMT:SJP | 2 | Some concerns | Low risk | No concerns | Major concerns | No concerns | No concerns | Very low | ["Within-study bias","Imprecision"] |
| NTKKNQ:SJP | 1 | Major concerns | Low risk | No concerns | Major concerns | No concerns | No concerns | Very low | ["Within-study bias","Imprecision"] |
| SJP:SJP_Ab | 2 | No concerns | Low risk | No concerns | Major concerns | No concerns | No concerns | Low | ["Imprecision"] |
| Ab:FFSW | 0 | No concerns | Low risk | No concerns | Major concerns | No concerns | No concerns | Low | ["Imprecision"] |
| Ab:NMT | 0 | No concerns | Low risk | No concerns | Major concerns | No concerns | No concerns | Low | ["Imprecision"] |
| Ab:NTKKNQ | 0 | Some concerns | Low risk | No concerns | Major concerns | No concerns | No concerns | Very low | ["Within-study bias","Imprecision"] |
| BXFQ_Ab:FFSW | 0 | Some concerns | Low risk | No concerns | Major concerns | No concerns | No concerns | Very low | ["Within-study bias","Imprecision"] |
| BXFQ_Ab:NMT | 0 | Some concerns | Low risk | No concerns | Major concerns | No concerns | No concerns | Very low | ["Within-study bias","Imprecision"] |
| BXFQ_Ab:NMT_Ab | 0 | Some concerns | Low risk | No concerns | Major concerns | No concerns | No concerns | Very low | ["Within-study bias","Imprecision"] |
| BXFQ_Ab:NTKKNQ | 0 | Some concerns | Low risk | No concerns | Major concerns | No concerns | No concerns | Very low | ["Within-study bias","Imprecision"] |
| BXFQ_Ab:SD_Ab | 0 | Some concerns | Low risk | No concerns | Major concerns | No concerns | No concerns | Very low | ["Within-study bias","Imprecision"] |
| BXFQ_Ab:SJP | 0 | No concerns | Low risk | No concerns | Major concerns | No concerns | No concerns | Low | ["Imprecision"] |
| BXFQ_Ab:SJP_Ab | 0 | Some concerns | Low risk | No concerns | Major concerns | No concerns | No concerns | Very low | ["Within-study bias","Imprecision"] |
| BXFQ_Ab:XNA_Ab | 0 | Some concerns | Low risk | No concerns | Major concerns | No concerns | No concerns | Very low | ["Within-study bias","Imprecision"] |
| BXFQ_Ab:YHMYL_Ab | 0 | Some concerns | Low risk | No concerns | Major concerns | No concerns | No concerns | Very low | ["Within-study bias","Imprecision"] |
| FFSW:NMT | 0 | Some concerns | Low risk | No concerns | Major concerns | No concerns | No concerns | Very low | ["Within-study bias","Imprecision"] |
| FFSW:NMT_Ab | 0 | Some concerns | Low risk | No concerns | Major concerns | No concerns | No concerns | Very low | ["Within-study bias","Imprecision"] |
| FFSW:NTKKNQ | 0 | Major concerns | Low risk | No concerns | Major concerns | No concerns | No concerns | Very low | ["Within-study bias","Imprecision"] |
| FFSW:SD_Ab | 0 | Some concerns | Low risk | No concerns | Major concerns | No concerns | No concerns | Very low | ["Within-study bias","Imprecision"] |
| FFSW:SJP_Ab | 0 | No concerns | Low risk | No concerns | Major concerns | No concerns | No concerns | Low | ["Imprecision"] |
| FFSW:XNA_Ab | 0 | Some concerns | Low risk | No concerns | Major concerns | No concerns | No concerns | Very low | ["Within-study bias","Imprecision"] |
| FFSW:YHMYL_Ab | 0 | Some concerns | Low risk | No concerns | Major concerns | No concerns | No concerns | Very low | ["Within-study bias","Imprecision"] |
| NMT:NMT_Ab | 0 | Some concerns | Low risk | No concerns | Major concerns | No concerns | No concerns | Very low | ["Within-study bias","Imprecision"] |
| NMT:NTKKNQ | 0 | Major concerns | Low risk | No concerns | Major concerns | No concerns | No concerns | Very low | ["Within-study bias","Imprecision"] |
| NMT:SD_Ab | 0 | Some concerns | Low risk | No concerns | Major concerns | No concerns | No concerns | Very low | ["Within-study bias","Imprecision"] |
| NMT:SJP_Ab | 0 | No concerns | Low risk | No concerns | Major concerns | No concerns | No concerns | Low | ["Imprecision"] |
| NMT:XNA_Ab | 0 | Some concerns | Low risk | No concerns | Major concerns | No concerns | No concerns | Very low | ["Within-study bias","Imprecision"] |
| NMT:YHMYL_Ab | 0 | Some concerns | Low risk | No concerns | Major concerns | No concerns | No concerns | Very low | ["Within-study bias","Imprecision"] |
| NMT_Ab:NTKKNQ | 0 | Some concerns | Low risk | No concerns | Major concerns | No concerns | No concerns | Very low | ["Within-study bias","Imprecision"] |
| NMT_Ab:SD_Ab | 0 | Some concerns | Low risk | No concerns | Major concerns | No concerns | No concerns | Very low | ["Within-study bias","Imprecision"] |
| NMT_Ab:SJP | 0 | No concerns | Low risk | No concerns | No concerns | Major concerns | No concerns | Low | ["Heterogeneity"] |
| NMT_Ab:SJP_Ab | 0 | Some concerns | Low risk | No concerns | Major concerns | No concerns | No concerns | Very low | ["Within-study bias","Imprecision"] |
| NMT_Ab:XNA_Ab | 0 | Some concerns | Low risk | No concerns | Major concerns | No concerns | No concerns | Very low | ["Within-study bias","Imprecision"] |
| NMT_Ab:YHMYL_Ab | 0 | Some concerns | Low risk | No concerns | Major concerns | No concerns | No concerns | Very low | ["Within-study bias","Imprecision"] |
| NTKKNQ:SD_Ab | 0 | Some concerns | Low risk | No concerns | Major concerns | No concerns | No concerns | Very low | ["Within-study bias","Imprecision"] |
| NTKKNQ:SJP_Ab | 0 | Some concerns | Low risk | No concerns | Major concerns | No concerns | No concerns | Very low | ["Within-study bias","Imprecision"] |
| NTKKNQ:XNA_Ab | 0 | Some concerns | Low risk | No concerns | Major concerns | No concerns | No concerns | Very low | ["Within-study bias","Imprecision"] |
| NTKKNQ:YHMYL_Ab | 0 | Some concerns | Low risk | No concerns | Major concerns | No concerns | No concerns | Very low | ["Within-study bias","Imprecision"] |
| SD_Ab:SJP | 0 | No concerns | Low risk | No concerns | Major concerns | No concerns | No concerns | Low | ["Imprecision"] |
| SD_Ab:SJP_Ab | 0 | Some concerns | Low risk | No concerns | Major concerns | No concerns | No concerns | Very low | ["Within-study bias","Imprecision"] |
| SD_Ab:XNA_Ab | 0 | Some concerns | Low risk | No concerns | Major concerns | No concerns | No concerns | Very low | ["Within-study bias","Imprecision"] |
| SD_Ab:YHMYL_Ab | 0 | Some concerns | Low risk | No concerns | Major concerns | No concerns | No concerns | Very low | ["Within-study bias","Imprecision"] |
| SJP:XNA_Ab | 0 | No concerns | Low risk | No concerns | Major concerns | No concerns | No concerns | Low | ["Imprecision"] |
| SJP:YHMYL_Ab | 0 | No concerns | Low risk | No concerns | Major concerns | No concerns | No concerns | Low | ["Imprecision"] |
| SJP_Ab:XNA_Ab | 0 | Some concerns | Low risk | No concerns | Major concerns | No concerns | No concerns | Very low | ["Within-study bias","Imprecision"] |
| SJP_Ab:YHMYL_Ab | 0 | Some concerns | Low risk | No concerns | Major concerns | No concerns | No concerns | Very low | ["Within-study bias","Imprecision"] |
| XNA_Ab:YHMYL_Ab | 0 | Some concerns | Low risk | No concerns | Major concerns | No concerns | No concerns | Very low | ["Within-study bias","Imprecision"] |

Appendix C.

|  | Ab | BXFQ_Ab | LQ | NMT_Ab | SJP | SJP_Ab | XNA_Ab |
| --- | --- | --- | --- | --- | --- | --- | --- |
| Ab | Ab | 1.09 (0.42, 2.89) | 0.67 (0.26, 1.7) | 1.62 (0.57, 4.8) | 1.16 (0.62, 2.62) | 0.68 (0.31, 1.62) | 1.46 (0.86, 2.51) |
| BXFQ_Ab | 0.91 (0.35, 2.41) | BXFQ_Ab | 0.61 (0.16, 2.36) | 1.49 (0.35, 6.22) | 1.06 (0.35, 3.94) | 0.61 (0.19, 2.36) | 1.34 (0.44, 4.04) |
| LQ | 1.49 (0.59, 3.79) | 1.64 (0.42, 6.27) | LQ | 2.42 (0.6, 10.03) | 1.72 (0.59, 6.13) | 1 (0.32, 3.72) | 2.18 (0.76, 6.4) |
| NMT_Ab | 0.62 (0.21, 1.76) | 0.67 (0.16, 2.84) | 0.41 (0.1, 1.66) | NMT_Ab | 0.72 (0.21, 2.78) | 0.42 (0.12, 1.64) | 0.9 (0.27, 2.93) |
| SJP | 0.86 (0.38, 1.6) | 0.95 (0.25, 2.85) | 0.58 (0.16, 1.7) | 1.39 (0.36, 4.69) | SJP | 0.58 (0.26, 1.24) | 1.27 (0.47, 2.84) |
| SJP_Ab | 1.48 (0.62, 3.18) | 1.64 (0.42, 5.35) | 1 (0.27, 3.15) | 2.41 (0.61, 8.67) | 1.73 (0.81, 3.88) | SJP_Ab | 2.18 (0.76, 5.43) |
| XNA_Ab | 0.68 (0.4, 1.16) | 0.75 (0.25, 2.25) | 0.46 (0.16, 1.32) | 1.11 (0.34, 3.71) | 0.79 (0.35, 2.12) | 0.46 (0.18, 1.31) | XNA_Ab |

Appendix D. Inconsistent test

Fig.D.1. Inconsistent test for cure rate


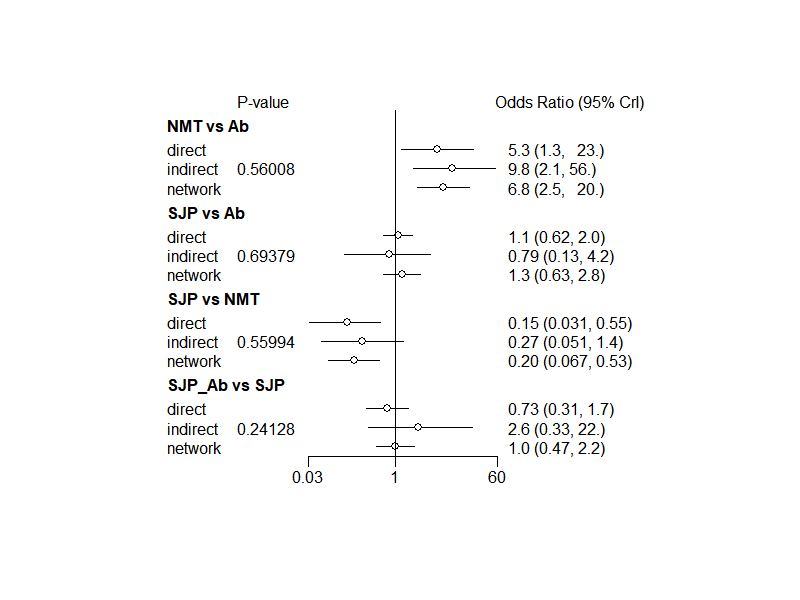


Fig.D.2. Inconsistent test for effective rate


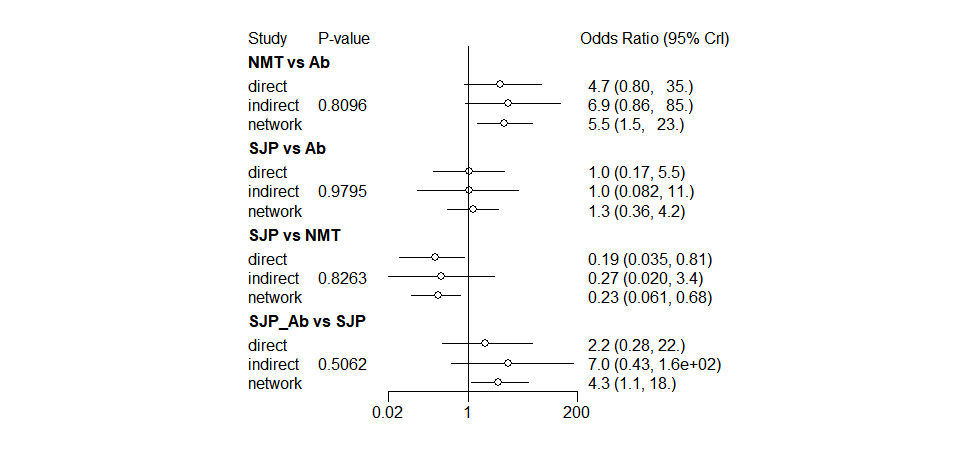


Appendix E: convergence diagnostics plot and trace and density plot

Fig.E.1. convergence diagnostics plot for cure rate


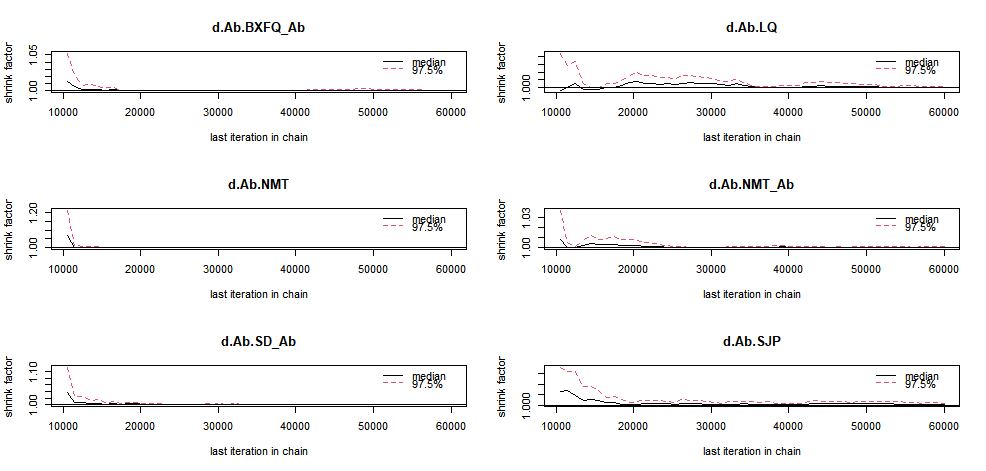


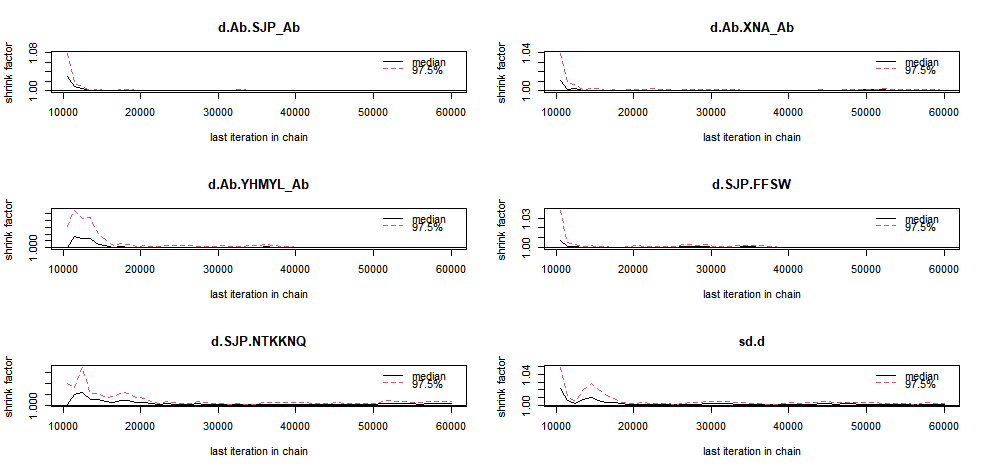


Fig.E.2. trace and density plot for cure rate


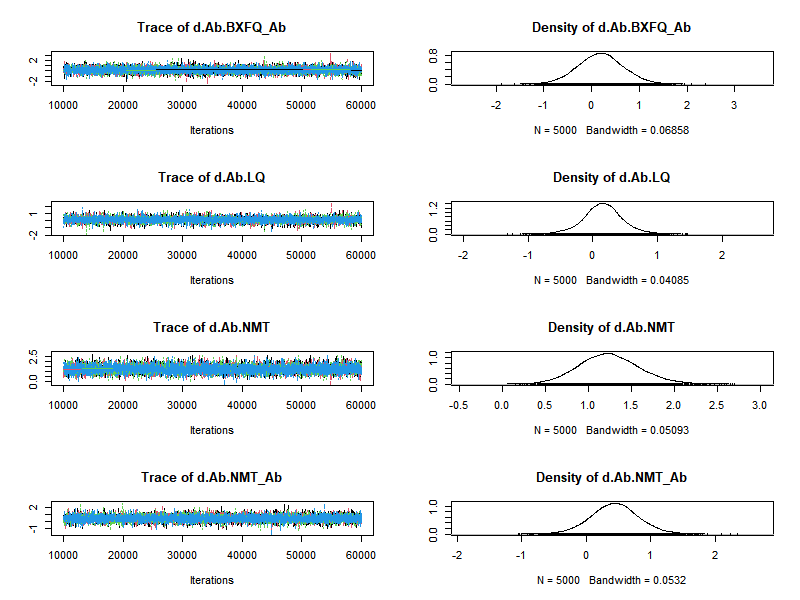


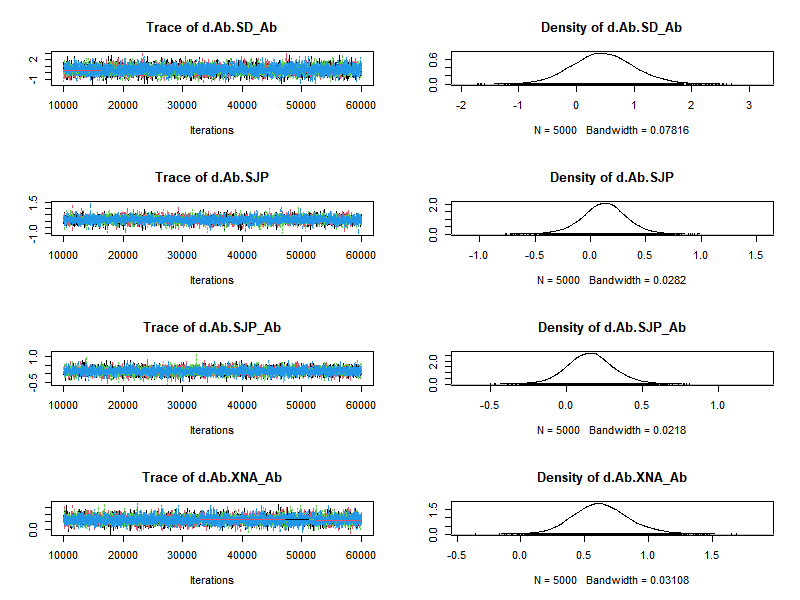


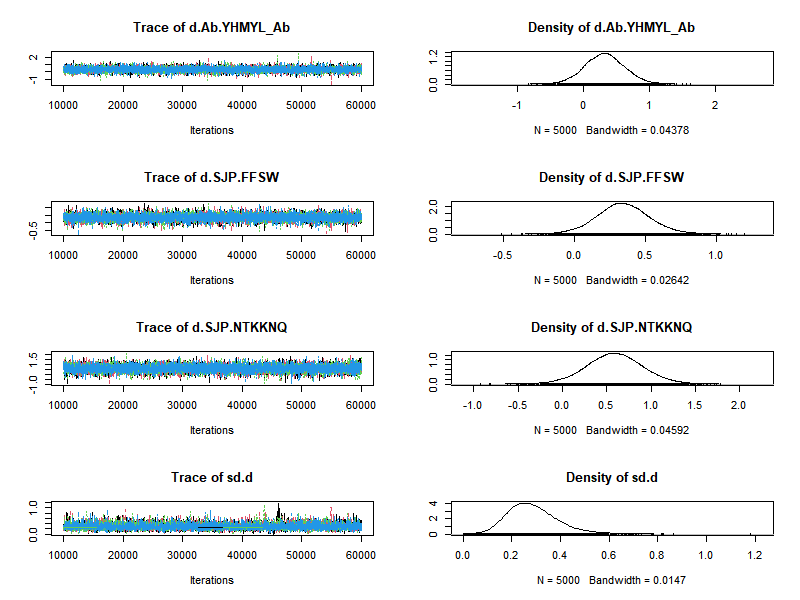


Fig.E.3. convergence diagnostics plot for effective rate


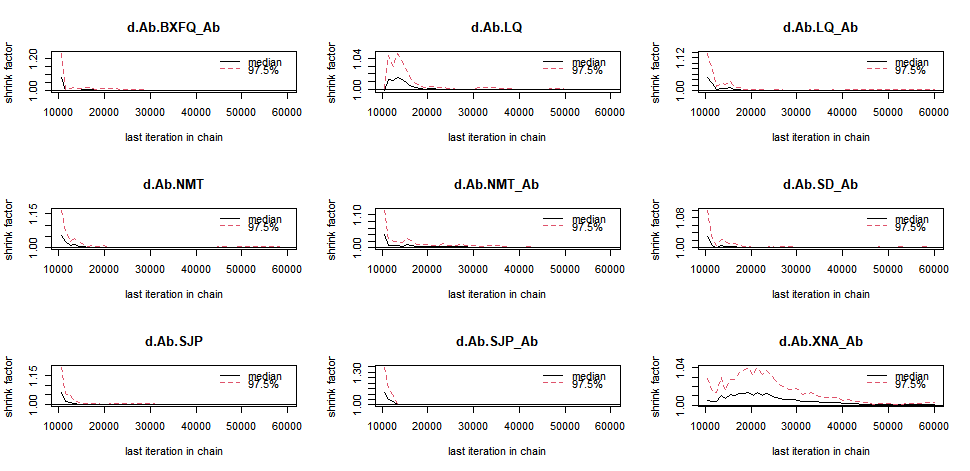


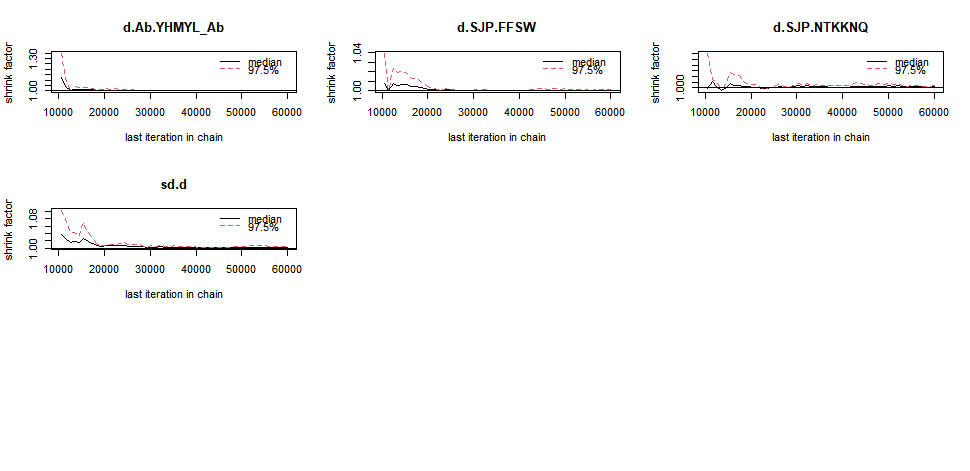


Fig.E.4. trace and density plot for effective rate


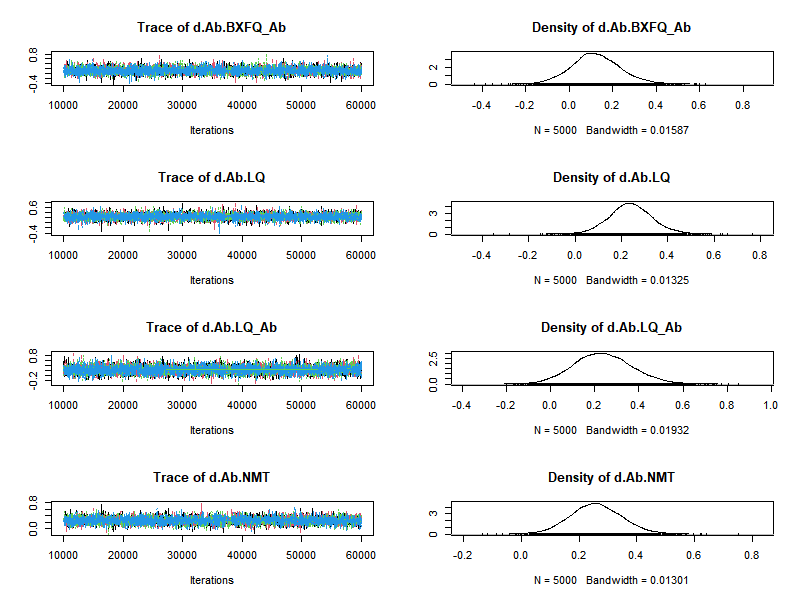


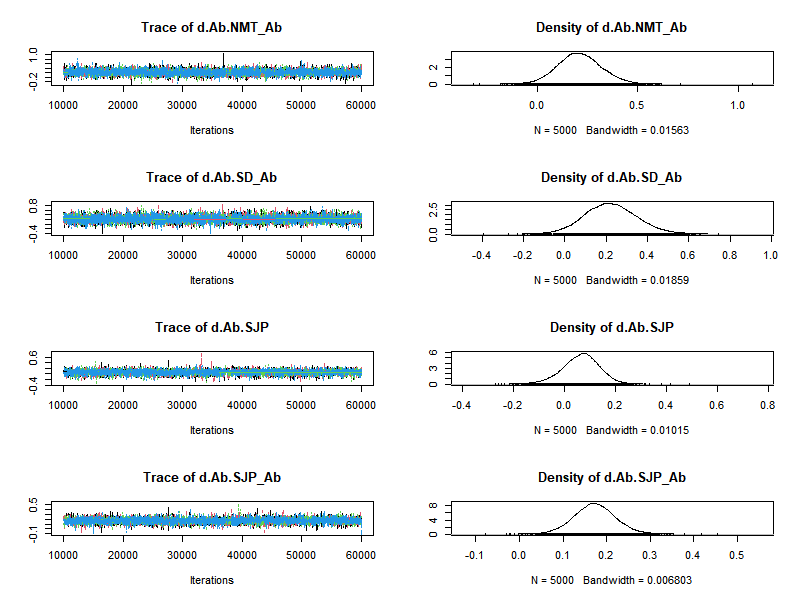


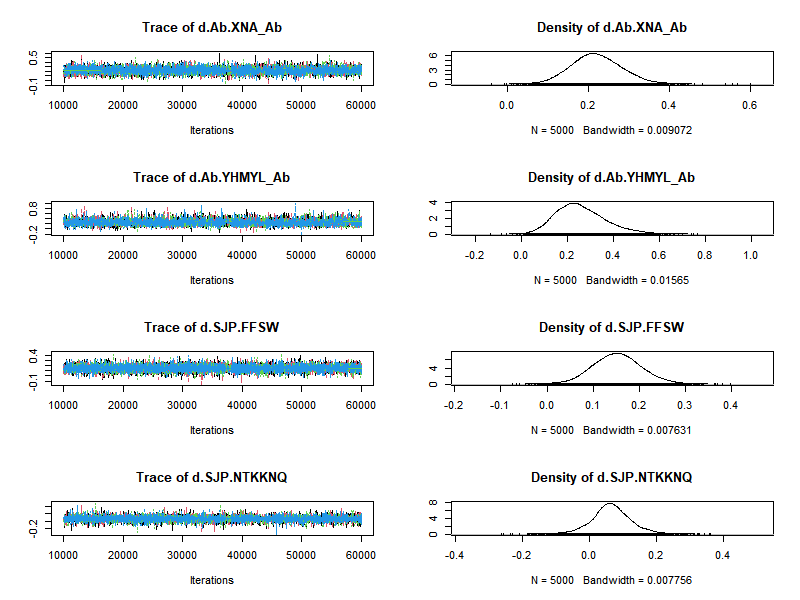


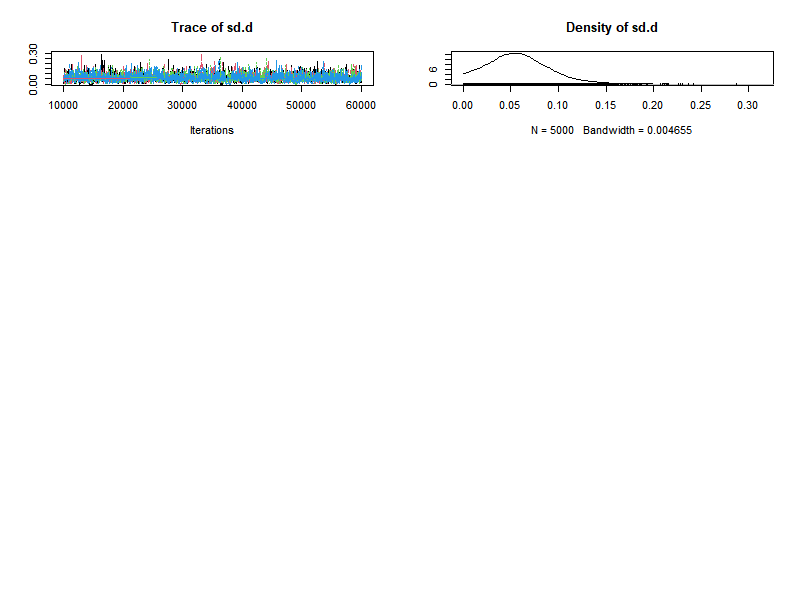


Fig.E.5. convergence diagnostics plot for bacterial clearance


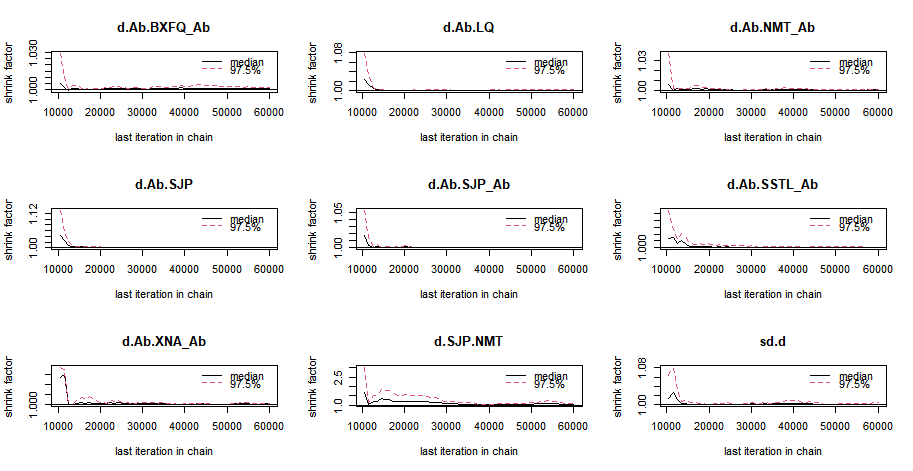


Fig.E.6. trace and density plot forbacterial clearance


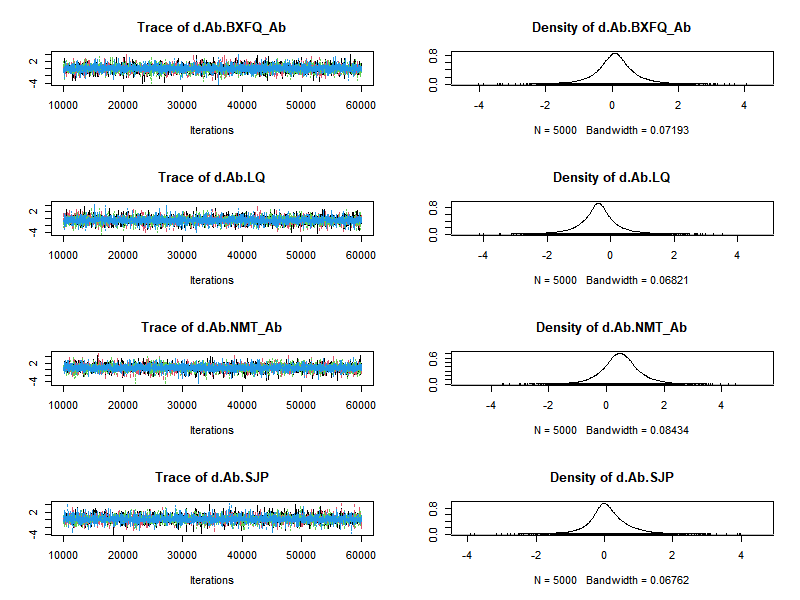


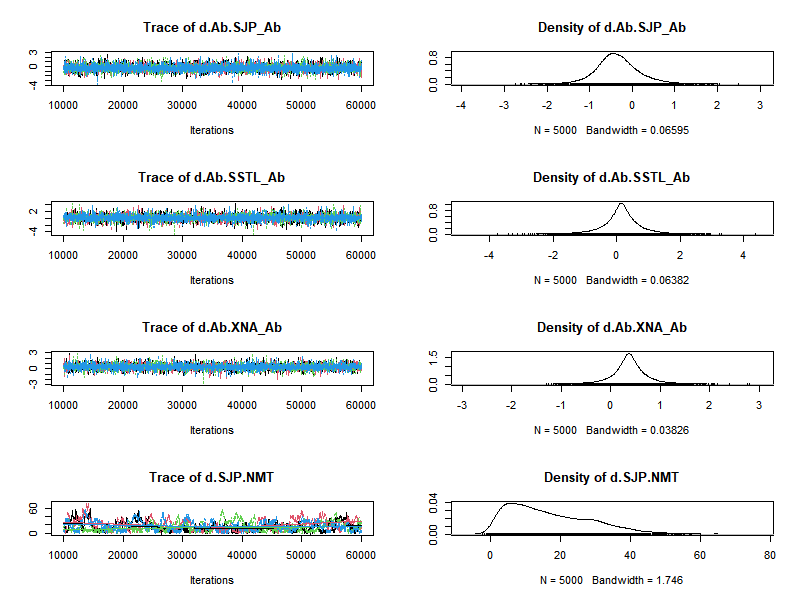


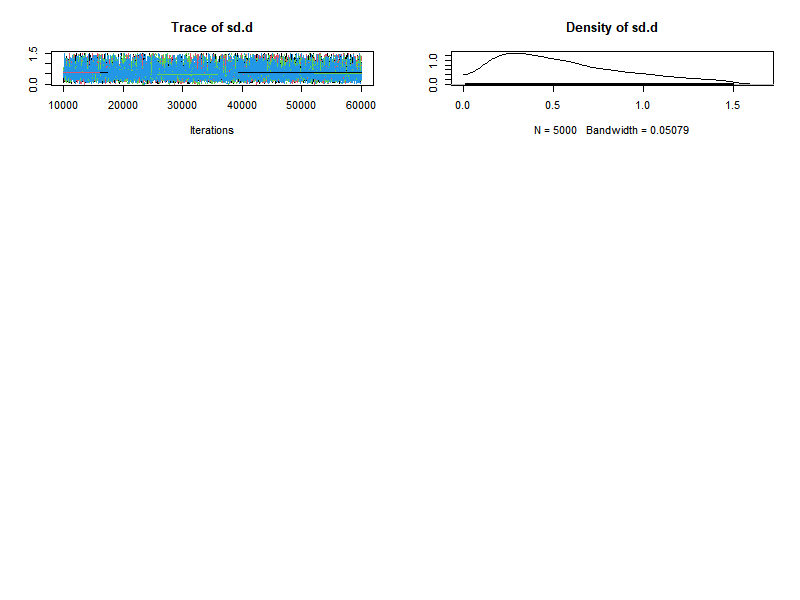


Fig.E.7. convergence diagnostics plot for adverse events


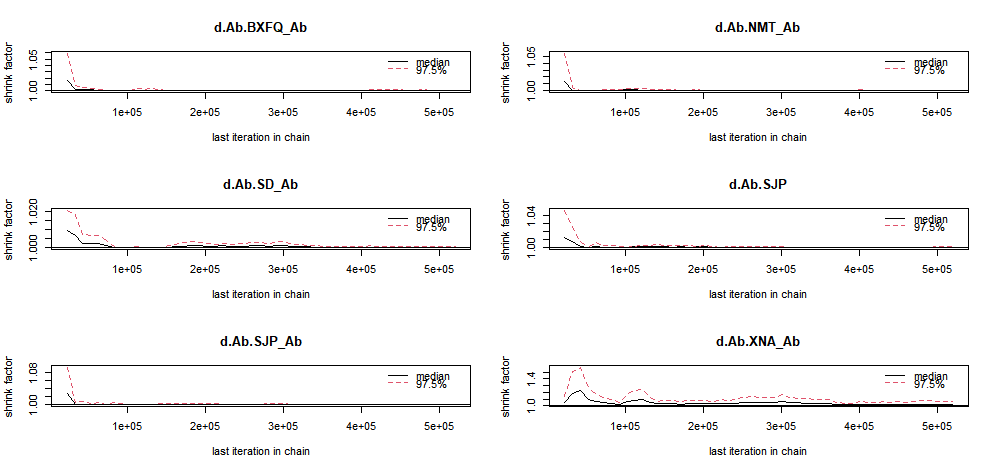


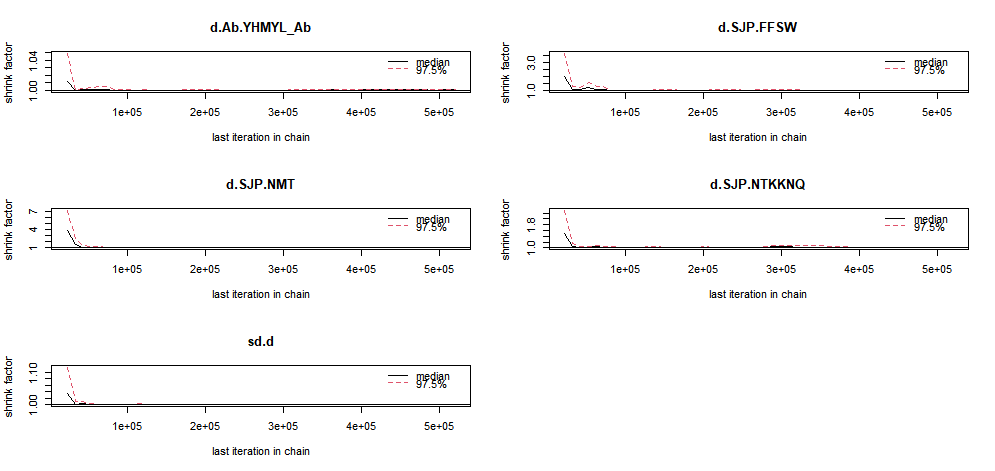


Fig.E.8. trace and density plot for adverse events


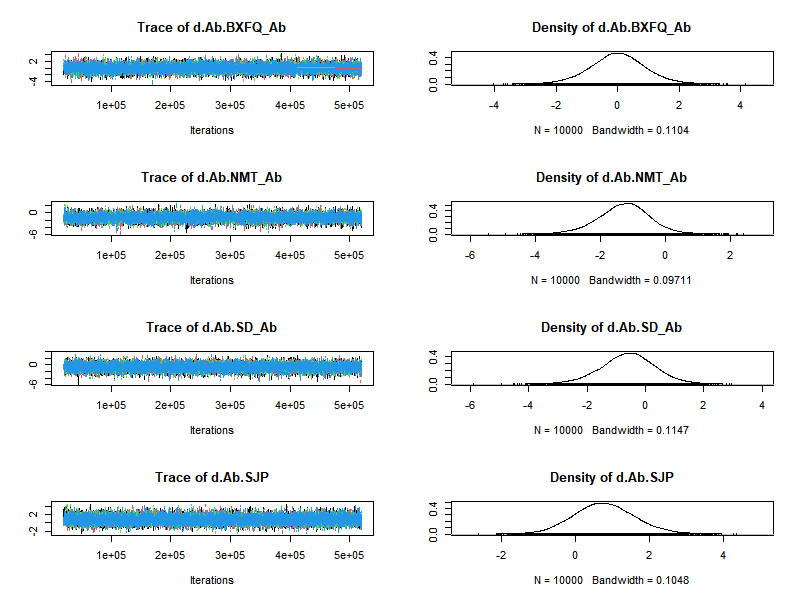


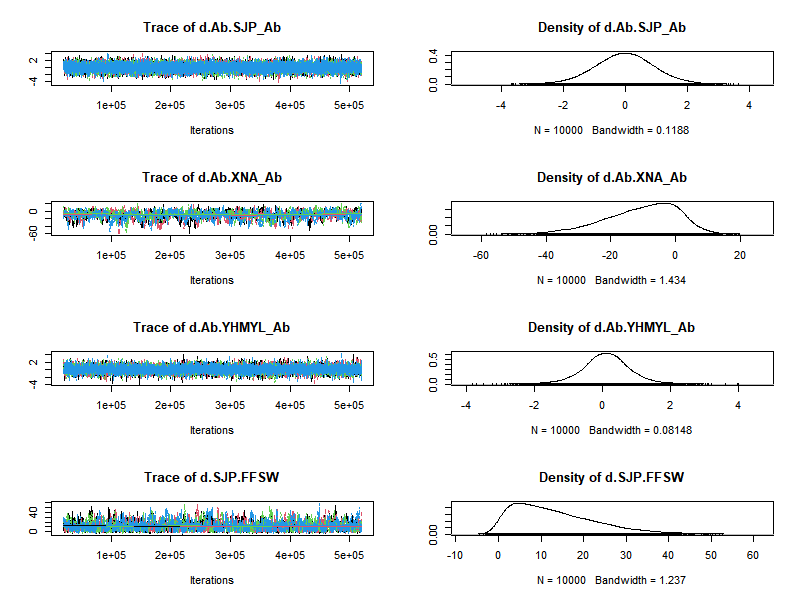


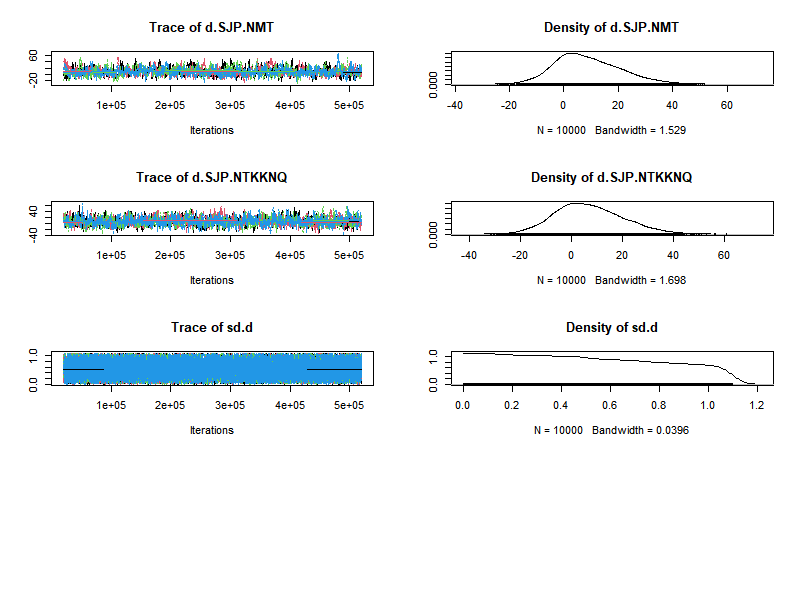


Abbreviations: BXFQ_Ab: Bixiefenqing pill combined with antibiotics; FFSW: Compound Shiwei tablet; LQ: Longqing tablet; LQ_Ab: Longqing tablet combined with antibiotics; NMT: Ningmitai capsule; NMT_Ab: Ningmitai capsule combined with antibiotics; NTKKNQ: Niaotongkakenaiqi tablet; SD_Ab: Shuangdong capsule combined with antibiotics; SJT: Sanjin tablet; SJT_Ab: Sanjin tablet combined with antibiotics; XNA_Ab: Xueniaoan capsule combined with antibiotics; YHMYL_Ab: Yinhua Miyanling tablet combined with antibiotics; Ab: conventional antibiotis.
